# Direct coupling of CO_2_ with epoxides catalyzed by lanthanum(III) supported on magnetic mesoporous organosilica nanoparticles

**DOI:** 10.1038/s41598-023-32647-9

**Published:** 2023-04-04

**Authors:** Kosar Sadat Hoseini, Masoumeh Razaghi, Tohid Nouri, Mojtaba Khorasani

**Affiliations:** 1 Department of Chemistry, Institute for Advanced Studies in Basic Sciences (IASBS), No. 444, Prof. Yousef Sobouti Boulevard, Zanjan, 45137-66731 Iran; 2 Research Center for Basic Sciences & Modern Technologies (RBST), Institute for Advanced Studies in Basic Sciences (IASBS), Zanjan, 45137-66731 Iran

**Keywords:** Catalysis, Heterogeneous catalysis

## Abstract

Lanthanum(III) supported on the magnetic mesoporous organosilica nanoparticle (La@MON) has been described as an efficient, simple, and durable heterogeneous catalyst for the synthesis of 5-membered cyclic carbonates from carbon dioxide (CO_2_) and epoxides. Under optimized reaction conditions, various terminal epoxides have been converted to the corresponding carbonates in the presence of 0.3 mol% La@MON and 0.5 mol% tetrabutylammonium iodide (TBAI) as co-catalyst at relatively mild reaction conditions. It was also found that La@MON catalysts had significantly higher catalytic activity than some selected reference catalysts, which can be explained by the abundance of lanthanum(III) species acting as Lewis acidic sites for activating both carbon dioxide and epoxide molecules, along with the fact that the catalyst channels are short and provided facile mass transfer. The catalyst showed good reusability for at least five reaction cycles while the magnetic core of the catalyst helps the easy separation of the catalyst by just using an external magnet.

## Introduction

Fine chemical synthesis by using carbon dioxide (CO_2_) as a readily available, cheap, nontoxic, and versatile C1 building block is a very attractive but challenging transformation from both synthetic and industrial points of view^1,2^. As an example, cyclic carbonates which have been extensively used as battery electrolytes, pharmaceutical, polymer, and engineering plastic syntheses, and polar aprotic solvents, can be obtained in 100% atom-economic reaction from the direct coupling of carbon dioxide and epoxides^3^. However, because of CO_2_ is thermodynamically stable and chemically inert, its utilization would be practically needed an active catalyst to avoid high temperatures and CO_2_ pressure^4^. In this regard, a substantial amount of research has been conducted to develop new and efficient heterogeneous and recyclable catalysts that can efficiently achieve CO_2_ cycloaddition to epoxides to form desirable five-membered cyclic carbonates in light of stringent environmental issues, green chemistry, and especially atom efficiency^5–10^. Most Recently, the synthesis of cyclic carbonates via CO_2_/Epoxides coupling catalyzed using sustainable catalytic systems under ambient conditions (1–5 bar and temp. up to 80 °C) or more specifically recovery heterogeneous catalysts under mild reaction conditions have been reported^4,11–13^.

While main and transition metal groups have been extensively explored^14–24^, rare-earth metals such as La(III) were rarely investigated for direct cycloaddition of CO_2_ to epoxides^25–27^. The high electron charge of the La(III) cation, the large ion radius, excellent oxophilicity, and the abundant outer hybrid orbitals, make La(III) species an excellent Lewis acidic catalyst for any organic reactions that polarization of C–O bond would be demanded^28^. The La(III) complexes are also known as powerful candidates for the activation of CO_2_ molecule^29^. It is well-documented that due to the efficient capture of CO_2_ via its reversible insertion into La(III) complexes, this vital transformation not only can be even performed under ambient conditions but also exhibit promising results in terms of activity and selectivity for the preparation of 5-membered cyclic carbonate^30^. For example, the Lanthanum complex tagged by ammonium iodide was found to be an efficient catalyst for converting terminal epoxides into cyclic carbonates in moderate to excellent yields at 40 °C under 1 bar CO_2_^31^. Atmospheric chemical fixation of CO_2_ by zinc-rare earth metal (Zn–RE) heterometallic complexes was independently reported by Yao and Liu and coworkers^32–35^. Okuda and Mashima et al. reported heteronuclear complexes of RE–Zn supported by macrocyclic tris(salen)-based ligand for alternating copolymerization of epoxide and CO_2_^36^. Castro-Osma and Lara-Sánchez et al. have described the synthesis of bio-derived furan- and diacid-derived cyclic carbonates in the presence of bis(silylamide) lanthanum complex as catalyst^37^. Although the above homogeneous lanthanum catalysts exhibit excellent activity and selectivity, suffer from less reusability. This issue makes more important when extensive ligand or lanthanum precursor was used. Nevertheless, heterogenized lanthanum-based catalysts have been rarely studied for carbon dioxide fixation to cyclic carbonate. Along this line, despite some reports on the recoverable lanthanum or even lanthanide catalysts based on metal–organic framework structures, ordered mesoporous silicas/organosilicas have been rarely used for the immobilization of these efficient catalysts^38–42^. To the best of our knowledge, there is just one example in which using a large-pore dehydrate ordered mesoporous silica (SBA-15) modified by cerium and lanthanum pyrazolate complexes, Anwander et al. found that 0.5 mol% rare-earth metal catalyst, 0.5 mol% tetrabutylammonium bromide (TBAB) and 10 bar CO_2_ could act as a recoverable catalyst for 5-membered carbonate synthesis from carbon dioxide under 90 °C for 24 h^43^.

In recent years, mesoporous organosilica materials, as a type of porous organic–inorganic hybrids, have received considerable attention because of their excellent physicochemical characteristics including high porosity and specific surface area, and adjustable pore size, which makes them ideal candidates in a wide variety of fields, from gas separation and targeted drug delivery to the design of smart catalysts^44–48^. It is well-documented that when these types of materials are designed in nanoparticle morphology would not only provide selective functionalization of those outer and inner surfaces for the design of cooperative catalytic systems but also can be used to enhance the activity of immobilized catalysts due to fast mass transfer within the short channels of catalyst^49–51^. However, due to the difficulty of separation, which usually requires ultracentrifugation, practical applications of mesoporous organosilica nanoparticles have been limited^52^. To address this limitation, by deposition of a layer of mesoporous silica/organosilica bearing catalyst on the magnetic nanoparticle as core, a porous core–shell catalyst can be obtained^53,54^. Magnetic core provides the possibility for simple separation of catalyst by using an external magnet while a thin mesoporous shell meets a good chance for mass transfer of reaction mixture^55^. However, magnetic MONs have rarely been investigated in the design of catalytic systems for various organic transformations, despite their many applications in nanomedicine^56^. To the best of our knowledge, there is no example of the use of lanthanum species supported by the mesoporous organosilica nanoparticle (MON) for the preparation of cyclic carbonate through CO_2_ cycloaddition to the epoxides^9^.

**Figure 1:**
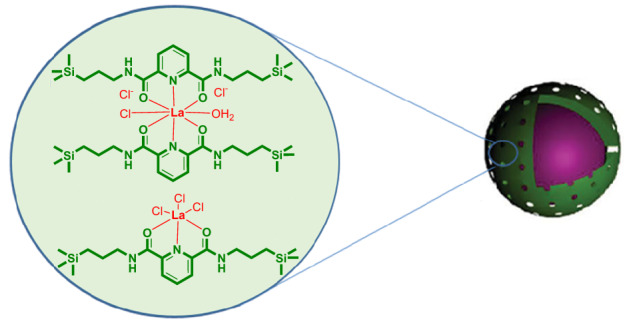
Schematically representation of La@MON.

We have recently described that hollow sphere mesoporous silica (HMS) through a confinement effect could surprisingly enhance the activity of tetraalkylammonium halide during the coupling of carbon dioxide with epoxides^57^. Although the HMS exhibited high activity due to short mesoporous channels, inherently suffers from difficulty in separation from the reaction medium. On the other hand, it has been shown that dipicolinic carboxamide incorporated in the pore walls of periodic mesoporous organosilica nanoparticles can be considered an excellent solid ligand for lanthanide groups due to their oxophilicity^58,59^. Herein, considering Lewis acidic nature of lanthanum species and enhancement of retention time of gas molecules in the porous organosilica framework as well as magnetically recoverability, we wish to disclose La(III) on the magnetic mesoporous organosilica nanoparticle (La@MON) with the pyridine-2,6-dicarboxamide framework (Fig. 1) in the combination with tetrabutylammonium iodide as an efficient catalytic system for synthesis of cyclic carbonate under relatively mild reaction conditions.

## Results and discussion

The La@MON was synthesized with a two-step method in which Fe_3_O_4_ was used as both the core and magnetic parts. The monodispersed Fe_3_O_4_ nanoparticles were synthesized accordingly to Zhao’s report with slight modifications^60^. In the next step, organosilica precursor was synthesized by two-step procedures from a direct reaction of dipicolinic acid with thionyl chloride and followed by amide formation through the reaction of resulted intermediate with (3-aminopropyl)trimethoxysilane^59^. Then, a mesoporous organosilica shell was deposited on magnetic nanoparticles by using home-made dipicolinic organosilica pressures and tetraethylorthosilicate (TEOS) in the presence of cetyltrimethylammonium bromide (CTAB) as supramolecular structure directing agent under mild basic conditions. Finally, after removing CTAB by simple extraction, lanthanum species were immobilized into the MON channels through direct complexation of La^3+^ into dipicolinic carboxamide units^59^. The schematic of the La@MON catalyst was also depicted in Fig. 1.

**Figure 2:**
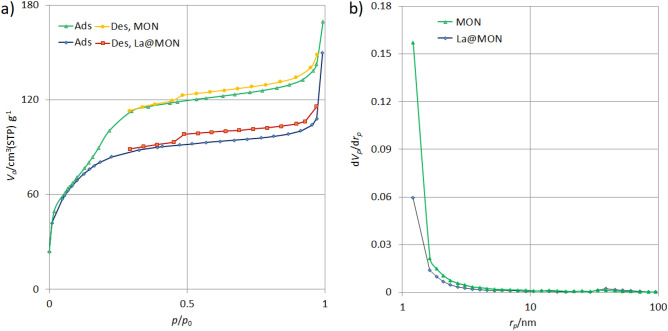
N_2_ adsorption–desorption isotherm (**a**) and BJH pore size distributions (**b**) for La@MON.

To gain more information about the surface area and porosity of the synthesized materials, the N_2_ adsorption/desorption isotherm was recorded at 77 K (Fig. 2a). Both MON and La@MON showed type IV isotherms with an H3 hysteresis loop according to the IUPAC classifications which are typical for materials with small mesopores^61^. The increase in N_2_ uptake in the higher relative pressure (~ 0.95) could be also defined as secondary porosity or inter-particle mesoporosity^62^. The BET (Brunauer–Emmett–Teller) specific surface area (S_BET_) and total pore volume (V_t_) for MON were found to be 312 m^2^ g^−1^ and 0.26 cm^3^ g^−1^, respectively (Table 1). After modification of MON with lanthanum(III) chloride, the amount of BET surface area and total pore volume were systematically decreased to 293 m^2^ g^−1^ and 0.23 cm^3^ g^−1^, respectively, a finding confirms the successful immobilization of lanthanum (III) in the catalyst pores. Since CTAB as structure directing agent was used, both MON and La@MON displayed a half-bell like BJH (Barrett-Joyner-Halenda) with relatively small pore size distributions had maxima (D_BJH_) at *ca.* 2.4 nm (Fig. 2b). The results overall confirm the mesoporous shell which provides the possibility for reactant diffusion and catalyst distribution was carefully deposited on the magnetic core.

**Table 1:** Textural properties of the synthesized materials were determined from nitrogen physisorption data.

Entry	Materials	S_BET_^a^ (m^2^ g^−1^)	V_t_^b^ (cm^3^ g^−1^)	D_BJH_^c^ (nm)
1	MON	312	0.26	2.4
2	La@MON	293	0.23	2.4

^a^S_BET_: Specific surface area was determined from the linear part of the BET in relative pressure from 0.05 to 0.15.

^b^V_t_: Total pore volume based on adsorbed N_2_ at P/P0 ≈ 0.995.

^c^D_BJH_: Pore size distribution calculated by BJH method from adsorption branch of nitrogen isotherm.

**Figure 3:**
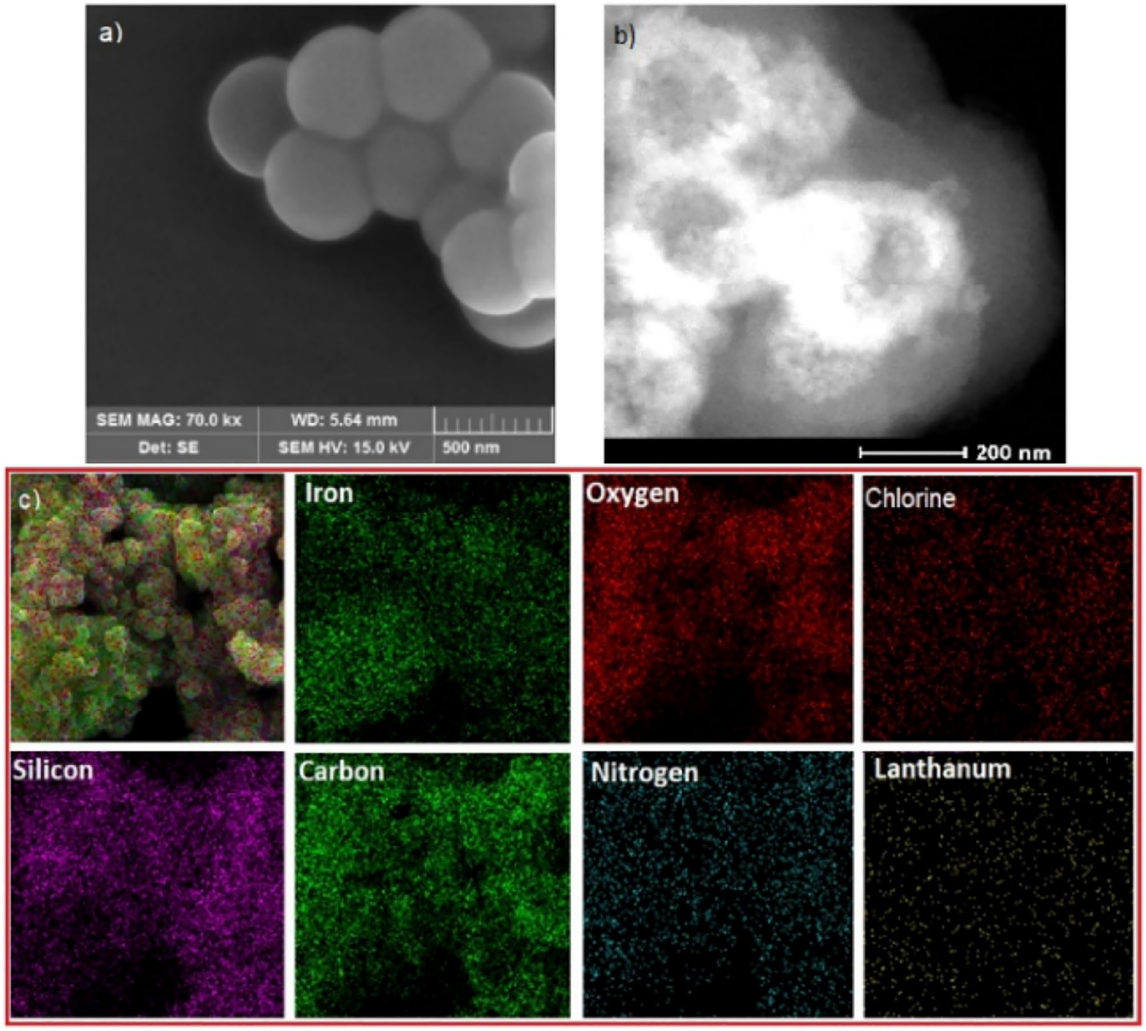
(**a**) SEM image (scale bar 500 nm), (**b**) TEM image (scale bar 200 nm), and (**c**) Energy dispersive X-ray spectroscopy (EDS) elemental maps for La@MON.

The scanning electron microscopy (SEM) image of La@MON showed monodispersed spherical nanoparticles with an estimated size of around 300 nm which is in good agreement with the results of the High-angle annular dark-field (HAADF) scanning transmission electron microscopy (STEM) image (Fig. 3a,b). To show the elemental distribution on La@MON, the energy dispersive X-ray spectroscopy (EDS) elemental maps from SEM image in high magnification was also recorded (Fig. 3c). As it is clear, all expected elements such as Fe, Si, O, C, N, Cl, and La were well-distributed in the sample.

**Figure 4:**
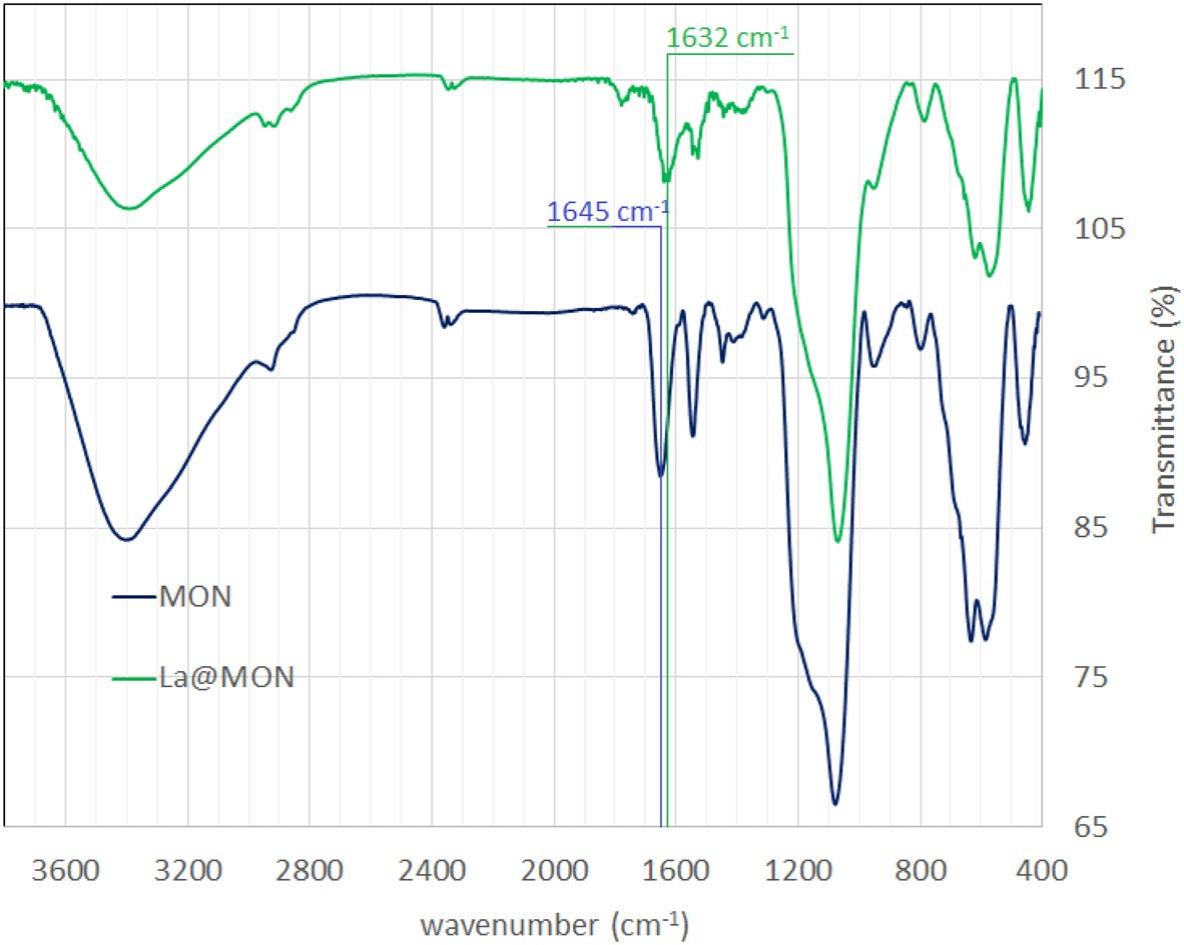
IR spectra of MON and La@MON.

The integrity of dipicolinic carboxamide units into the framework of MON and La@MON was confirmed by FTIR spectroscopy (Fig. 4). Because both samples have the same structure, very similar IR spectra were observed. The MON exhibited obvious vibration at 3060, 2870–2925 and 1655 cm^−1^ can be assigned to vibration of aromatic C–H, aliphatic C–H and amide bond, respectively^59^. The La@MON also displayed the related peaks with a red shift of the C=O vibration in the amide functional group (from 1645 to 1632, about 13 cm^−1^) which further demonstrated the successful incorporation of lanthanum in the DPA-Si molecular unit^59^.

**Figure 5:**
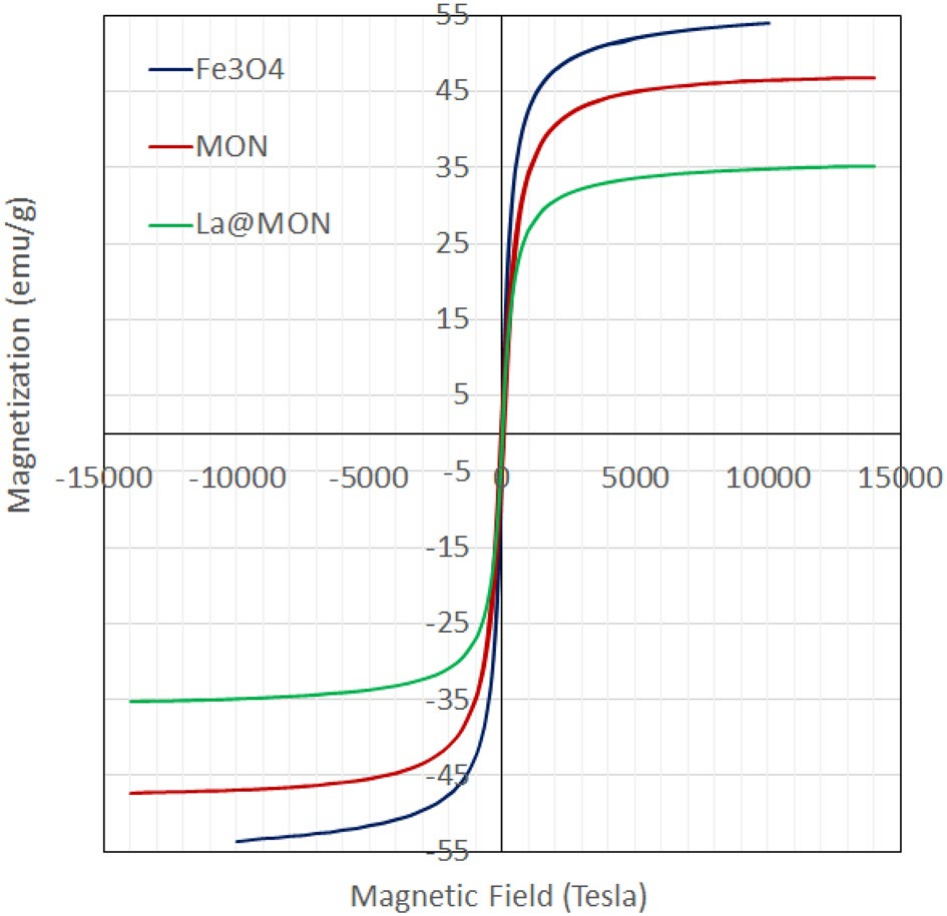
Field-dependent magnetization curves of Fe_3_O_4_, MON, and La@MON.

Magnetic measurements were performed by using a vibrating sample magnetometer (VSM) at 300 K (Fig. 5). Since there was no hysteresis in the magnetization for the magnetized nanoparticles as well as neither coercivity nor remanence, it can be speculated that all samples are superparamagnetic^63^. The decrease in saturation magnetization amount from Fe_3_O_4_ to La@MON might be responsible for the increased mass of mesoporous shell and lanthanum species deposited on the surface of magnetic cores. However, the La@MON still has good magnetic properties and could be easily and quickly removed from the reaction medium by exerting a magnet near the reaction vessels.

**Figure 6:**
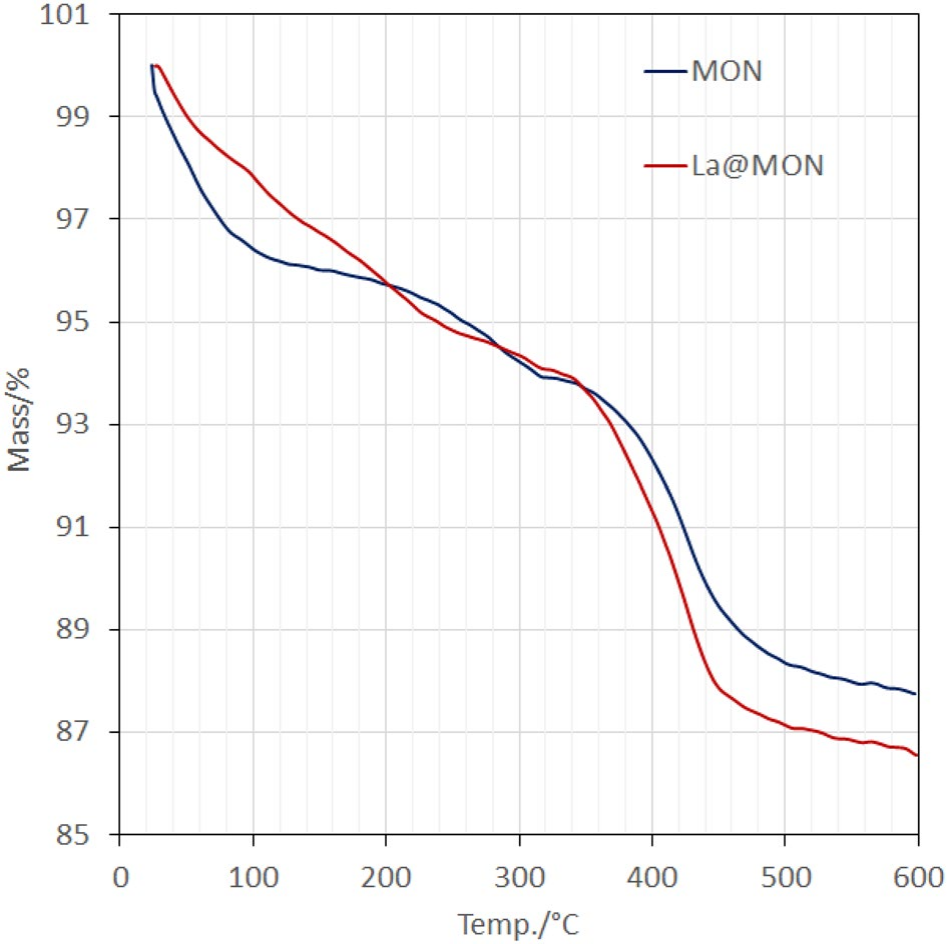
thermogravimetric patterns for MON and La@MON.

To evaluate the thermal stability and functional group loading for both MON and La@MON, the thermogravimetric analysis (TGA) was also performed over the temperature range of 25–600 °C with a temperature speed of 10 °C min^−1^ under an oxygen atmosphere (Fig. 6). As it is clear, all TGA patterns are very similar together and in overall both samples are thermally stable till 250 °C even under oxidizing atmosphere. The almost 4% weight loss under 200 °C is attributed to the removal of water and alcohol molecules from the pores of materials. The main weight loss ranging from 220 to 500 °C, can be assigned to the thermal decomposition of dipicolinic amide (denoted as ONO) units. Based on this result, the loading of ONO was found to be 0.32 mmol g^−1^ which is in good agreement with what was obtained from elemental analysis (CHN) (Table 2). Finally, the loading of lanthanum species was found to be 0.13 mmol g^−1^ by using Inductively coupled plasma mass spectrometry (ICP-MS) from acid-washed samples (Table 2).

**Table 2:** Estimation of functional group loading based on the elemental analysis and TG analyses.

Entry	Materials	%C	%N	TG weight lose (220–500 °C)	FG loading^a^ (mmol g^−1^)
1	MON	5.42	1.41	~ 8%	ONO (0.32)
2	La@MON	5.52	1.40	~ 9%	ONO (0.32), La (0.13)

^a^Loading of functional groups (lanthanum and dipicolinic carboxamide, ONO) calculated by ICP-MS, TGA, or elemental analysis (CHN).

Optimization studies on the performance of La@MON in the catalytic cycloaddition of CO_2_ to epoxides were conducted by using some tetraalkylammonium halides as co-catalyst and a variety of CO_2_ pressure and temperature under solvent-free reaction conditions (Table 3). All reaction conversions were calculated by using the gas chromatography technique while trimethylbenzene (TMB) was used as the internal standard. At first, the possibility of CO_2_ cycloaddition to the styrene oxide as substrate model was checked under 0.3 mol% La@MON, 0.5 mol% tetrabutylammonium iodide (TBAI), and 10 bar CO_2_ at different temperatures (Table 3, entries 1–4). While a low conversion of styrene oxide was obtained at 70 °C under described reaction conditions, a remarkable increase in styrene oxide conversion up to 92% could be observed at 100 °C (Table 3, entries 1–4). At 100 °C, the prolonging of reaction time till 12 h, just leads to a slight improvement of reaction conversion from 92 to 95% (Table 3, entry 4 vs. 5). The reaction conversion also remarkably dropped when either lower amount of La@MON (0.2 mol%) or TBAI (0.3 mol%) were used at 10 bar CO_2_ at 100 °C (Table 3, entries 6 and 7). The effect of CO_2_ pressure on the activity of the presented catalyst was also examined. Although the moderate conversion of styrene oxide was observed under 5 bar CO_2_, styrene oxide could be converted to styrene carbonate at 94 and 95% under 15 and 20 bar CO_2_, respectively (Table 3, entries 8–10). Despite the excellent results observed under higher CO_2_ pressure, because of safety reasons, we decided to use 10 bar CO_2_ for future studies. We also check the effect of higher loading of La@MON under milder conditions (low pressure and temperature), but no excellent results were observed (Table 3, entries 11–12). In the next step, we evaluated the activity of La@MON in the presence of another quaternary ammonium salt as a co-catalyst (Table 3, entries 13–15). Under the same reaction conditions, tetrabutylammonium bromide (TBAB) showed a relatively good reaction conversion of 75% (Table 3, entry 13). By using tetraethyl ammonium bromide (TEAB), the yield of styrene carbonate decreased to 55% (Table 3, entry 14). If tetrabutylammonium chloride (TBAC) was used, a low conversion of 33% was achieved under the same reaction conditions (Table 3, entry 15). The lower activity of TBAB, TEAB, and TBAC in comparison to TBAI can be related to the good ability of iodide ions in the ring opening of epoxide which is considered the rate-determining step of CO_2_ cycloaddition to epoxide^64^. We found that the use of 4-Dimethylaminopyridine (DMAP) as a co-catalyst just resulted in a poor yield of 13% (Table 3, entry 16). Due to the above results, it was established that the best result was obtained when the reaction was carried out in the presence of 0.3 mol% La@MON, 0.5 mol% TBAI, 10 bar pressure CO_2_ at 100 °C under solvent-free reaction conditions within 5 h (Table 3, entry 4). To highlight the role of lanthanum species and porous MON structures in obtaining high activity in the coupling of styrene oxide with carbon dioxide, some selected catalysts were also checked under optimized reaction conditions presented in entry 4 of Table 3. In the absence of La@MON and by employing TBAI alone, a low conversion of styrene oxide was attained (Table 3, entry 17). The use of 0.3 mol% Lanthanum chloride heptahydrate (LaCl_3_⋅H_2_O) in homogenous form (unsupported) instead of La@MON resulted in inferior yields of styrene carbonate (Table 3, entry 18). The higher catalytic activity of La@MON with regard to LaCl_3_⋅H_2_O may be attributed to the presence of dipicolinic amide unites incorporated inside of mesoporous channels of catalyst, which would favor the CO_2_ capturing through pyridine basic site during the reaction process as well as enchantment of metal center life time via its complexation on a solid network^43^. The studies also showed that the use of several selected catalysts such as MON, Fe_3_O_4_@mSiO_2,_ and Fe_3_O_4_ under the same reaction conditions and with essentially the same weight, resulted in very poor yields of styrene carbonate (Table 3, entries 19–21). As consequence, the presence of both lanthanum species and dipicolinic in the catalyst pores are crucial in getting high activity. We have also tried to experimentally show the affinity of pyridine's basic site to carbon dioxide according to the methods has already been reported by Anwander et al.^43^. To do this, a pre-weighted sample of La@MON and MON were stored under 1 bar CO_2_ pressure for 16 h at ambient temperature. After treatment, the calculations showed that 1.21 and 1.25 mmol CO_2_ per gram of La@MON and MON were captured, respectively. In a controlled experiment, under the same conditions, mesoporous silica-coated on the magnetic nanoparticle (Fe_3_O_4_@mSiO_2_) just exhibited 0.37 mmol CO_2_ g^−1^. These data confirmed the role of the basic pyridine site in the capture of carbon dioxide molecules. As the final part of optimization studies, we also prepared homogeneous lanthanum(III) catalyst and checked its activity under optimized reaction conditions. A homogeneous form of the catalyst denoted as HOM.La(III) leads to 78% conversion of styrene oxide with a TON of 260 while La@MON showed 92% conversion with a TON of 306 (Table 3, entry 22 vs. 4). These data also confirmed the crucial role of supported lanthanum catalysts in the meso-channels of MON for obtaining acceptable conversion and selectivity.

**Table 3:** Possibility for coupling of CO_2_ with styrene oxide under various reaction conditions.


Entry	Catalyst (mol%)	Additive (mol%)	t (h)	P_CO2_ (bar)	T (°C)	Conversion^a^ (%)	TON^b^
1	La@MON (0.3)	TBAI (0.5)	5	10	70	40	133
2	La@MON (0.3)	TBAI (0.5)	5	10	80	60	200
3	La@MON (0.3)	TBAI (0.5)	5	10	90	77	257
**4**	La@MON (0.3)	TBAI (0.5)	5	10	100	92	306
5	La@MON (0.3)	TBAI (0.5)	12	10	100	95	317
6	La@MON (0.3)	TBAI (0.3)	5	10	100	78	260
7	La@MON (0.2)	TBAI (0.5)	5	10	100	68	227
8	La@MON (0.3)	TBAI (0.5)	5	5	100	59	197
9	La@MON (0.3)	TBAI (0.5)	5	15	100	94	313
10	La@MON (0.3)	TBAI (0.5)	5	20	100	95	317
11	La@MON (0.6)	TBAI (0.5)	8	5	100	81	135
12	La@MON (0.6)	TBAI (0.5)	8	10	80	74	246
13	La@MON (0.3)	TBAB (0.5)	5	10	100	75	250
14	La@MON (0.3)	TEAB (0.5)	5	10	100	55	183
15	La@MON (0.3)	TBAC (0.5)	5	10	100	33	110
16	La@MON (0.3)	DMAP (0.5)	5	10	100	13	43
17	-	TBAI (0.5)	5	10	100	32	106^c^
18	LaCl_3_.7H_2_O (0.3)	TBAI (0.5)	5	10	100	58	193
19^d^	MON	TBAI (0.5)	5	10	100	54	180^c^
20^d^	Fe_3_O_4_@mSiO_2_	TBAI (0.5)	5	10	100	42	140^c^
21^d^	Fe_3_O_4_	TBAI (0.5)	5	10	100	35	117^c^
22^e^	Hom.La(III) (0.3)	TBAI (0.5)	5	10	100	78	260

Reaction conditions: styrene oxide (5 mmol), La@MON (115 mg, 0.3 mol% La to epoxide), and 10 bar CO_2_ unless otherwise specified.

^a^Conversion of styrene oxide determined by GC technique using trimethylbenzene as internal standard. Selectivity for styrene carbonate was consistently above 99%

^b^TON: turnover number calculated by [(mmol of carbonate)/(mmol of La)].

^c^TON was determined in terms of TBAI.

^d^115 mg of desired materials were used.

^e^Homogeneous complex of lanthanum(III) catalysts with N-butyl dipicolinic carboxamide.

La@MON catalyst was then explored for cycloaddition reaction of CO_2_ with a range of epoxides under optimized reaction conditions described in entry 4 of Table 3. It was found that terminal epoxides could be converted to the corresponding cyclic carbonates in good to excellent yields and selectivity, while internal epoxide such as cyclohexene oxide showed poor results (Table 4). It is notable to mention that, the selectivity for all reactions was determined to be almost > 99% by using gas chromatography techniques and trimethyl benzene as the internal standard. Since the short reaction time was obtained during optimization studies, we observe that there is no obvious fluctuation in conversions of terminal epoxides. Under optimized reaction conditions, the propylene oxide and butylene oxide were selectively transformed into the related cyclic carbonates in high conversion of 95 and 92%, respectively (Table 4, entries 2 and 3). Epichlorohydrin carbonate was obtained as the sole product with good results (Table 4, entry 4). Interestingly, in quantitative yield, glycidol could be converted to glycerol carbonate which is extensively used as an important chemical intermediate in pharmacy and industry (Table 4, entry 5). Alkyls and phenyl glycidyl ethers derivatives also showed good to excellent conversions under described reaction conditions (Table 4, entries 6–8). Butyl glycidyl ether exhibited relatively lower conversion in comparison to the same derivative which can be related to the difficulty of diffusion to the catalyst channels (Table 4, entry 7). In the case of isopropyl glycidyl ether, we found that a slightly higher reaction time was needed to achieve excellent results which may be related to the steric effect (Table 4, entry 7). By using the presented catalytic protocol, selective CO_2_ cycloaddition of both allyl glycidyl ether and glycidyl methacrylate derivatives was performed without any evidence for either carbon double bond oxidation or polymerization (Table 4, entries 9–10). Although cyclohexene oxide as a sluggish substrate just showed a low conversion of 26% in the presence of 0.3 mol% catalyst, the reaction yield could be improved up to 49% when 0.6 mol% of catalyst was used (Table 4, entries 11–12).

**Table 4:** Coupling of various epoxides with CO_2_ catalyzed by La@MON.


Entry	Epoxide	Time (h)	Conversion (%)^a^	TON^b^
1		5	92	306
2		5	95	317
3		5	92	306
4		5	87	290
5		5	99	327
6		5	71	237
7		8	94	313
8		5	95	317
9		5	95	317
10		5	95	317
11		24	26	86
12^c^		24	49	82

Reaction conditions: 5 mmol epoxides, 0.3 mol% La@MON, 0.5 mol% TBAI as co-catalyst, 10 bar CO_2_ at 100 °C under solvent-free conditions.

^a^All conversions were determined by the GC method with trimethylbenzene as the internal standard. All reaction selectivities were calculated to be more than 99%

^b^TON: turnover number calculated by [(mmol of carbonate)/(mmol of La)].

^c^0.6 mol % La@MON was used.

We also investigated the reusability of the La@MON during the coupling of styrene oxide and carbon dioxide as optimized reaction conditions described in entry 4 of Table 3. Due to the presence of Fe_3_O_4_ as a magnetic core, it is also possible to separate the La@MON catalyst from reaction mediums using an external magnet. The results showed that recycling the La@MON catalyst over five runs did not lead to a significant decline in styrene carbonate yields and selectivity (Table S1). In spite of several recycling steps under pressurized reaction conditions, no significant change in structural order or pore size distribution of the recovered catalyst was observed as a result of the N_2_ adsorption–desorption analysis (Figs. S1 and S2). For reused catalyst (Re-La@MON), the specific surface area, pore volume, and pore diameter were respectively 261 m^2^ g^−1^, 0.21 cm^3^ g^−1^, and 2.4 nm, which are very similar to fresh catalyst. It appears that the catalyst composition remains intact during catalyst recycling processes, according to FTIR and TGA results (Figs. S3 and S4). The SEM image of the recovered catalyst sample after the fifth run confirms that the LA@MON still remains monodisperse with a spherical shape (Fig. S5). The leaching of lanthanum species in reaction media and also after the latest catalyst recycling run were also evaluated. To do this, after the compellation of the reaction, the reaction mixture was collected in a falcon and the catalyst was separated by using an external magnet. The amount of La(III) in the supernatant was found to be < 2 ppm ICP-MS. After the fifth catalyst run, a sample of reused La@MON was also analyzed by ICP-MS to determine the La contents. The result showed the same loading for the metal catalyst as the fresh one (0.12 Vs. 0.13 mmol g^−1^) which is in good agreement with catalyst stability.

## Conclusion

In conclusion, novel magnetic mesoporous organosilica nanoparticles with pyridine carboxamide units have been described for immobilization of lanthanum(III) through ONO pincer complexation as a recoverable catalyst for the solvent-free cycloaddition reaction of CO_2_ with various epoxides to provide cyclic carbonates under relatively mild reaction conditions. Within short reaction times, different types of terminal epoxides from aliphatic to bearing sensitive functional groups were converted to their corresponding cyclic carbonates using 0.3 mol% La@MON, 0.5 mol% TBAI, and 10 bar CO_2_ at 100 °C. It is believed that the major reason for La@MON's high catalytic activity is the presence of dipicolinic amide units in mesoporous channels. In addition to capturing CO_2_ during the reaction process through the pyridine basic site, the complexation of the metal center on the solid network of MON would enhance the metal center's lifetime. The catalyst could be also recovered and reused for at least another four reaction cycles without any remarkable decrease in its activity or selectivity.

## Methods

### Synthesis of pyridine-2,6-dicarboximide organosilica precursor

The organosilica precursor was prepared by a previously reported method with slight modification^59^. In the first step, dipicolinic acid (2.5. g, 15 mmol) and thionyl chloride (25 ml, 345 mmol) were added into a flame-dried balloon and then refluxed for 15 h under argon. Then, the reaction mixture was cooled to ambient temperature, unreacted thionyl chloride was removed under reduced pressure. The solid residue as pyridine 2,6-dicarbonyl chloride was further dried in a vacuum oven overnight and utilized for the next step without any purification. In the second step, home-made pyridine 2,6-dicarbonyl chloride (0.5 g, 2.46 mmol) and dry THF (15 ml) were charged in a flame-dried balloon, then a mixture of (3-aminopropyl)trimethoxysilane (0.884 g, 0.866 ml, 4.93 mmol) and anhydrous pyridine (0.429 g, 0.436 ml, 5.42 mmol) in THF (5 ml) was added dropwise under argon. After having stirred at ambient temperature for 4 h, the reaction solution was filtered. THF and pyridine were removed by vacuum to obtain a yellow oil as pyridine-2,6-dicarboximide organosilica precursor.

### Synthesis magnetic mesoporous organosilica (MON)

In the first step, Fe_3_O_4_ as a magnetic core was synthesized according to Zhao’s report^60^. Then, 1 g of the as-prepared Fe_3_O_4_ was homogeneously dispersed in 200 mL of deionized water by ultrasonication for 15 min. The suspension was then added into a solution containing CTAB (1.5 g), deionized water (300 mL), ethanol (300 mL), and 28% ammonia solution (5.5 mL). A solution containing pre-synthesized organosilica precursor (308 mg, 0.62 mmol) and TEOS (3.11 ml, 14 mmol) in 3 ml ethanol was next added dropwise with stirring to the surfactant solution. The suspension was further stirred at room temperature for 6 h. The products were collected by an external magnet and washed several times with DI water and ethanol and dried at 80 °C overnight. To remove the CTAB template, 1 g of as-synthesized MON was stirred into a solution of ethanol (100 ml) and ammonium nitrate (0.2 ml) at 65 °C for 4 h. This process was repeated twice to completely remove all surfactants.

### Synthesis La(III) supported on magnetic mesoporous organosilica (La@MON)

In a typical procedure, to 30 mL of an ethanolic solution of LaCl_3_⋅7H_2_O (713 mg, 1.9 mmol), a fine powder of MON (1.7 g, 0.3 mmol ONO per g) was added and stirred at 80 °C for 15 h under argon. The product was collected by an external magnet, washed with ethanol, and dried at 80 °C to yield La@MON^59^.

### Catalytic conversion of CO_2_ and epoxide to the cyclic carbonate

Epoxide (5 mmol), tetrabutylammonium iodide (0.5 mol% with regard to the substrate), and La@MON (115, 0.3 mol% to epoxide) were added to a stainless steel high-pressure reactor. The reactor was then pressurized to 10 bar and the reaction mixture was stirred at 100 °C for the desired time. After the completion of the reaction, the reaction mixture was allowed to cool down to room temperature and a slow depressurization of the reactor was carried out. Followed by, 70 µL 1,3,5-trimethylbenzene (TMB) as internal standard and 5 mL ethyl acetate were added and the separation of the catalyst from the reaction mixture was easily performed by centrifugation. The supernatant solution was sampled and analyzed by gas chromatography. Then, the collected ethyl acetate was removed by reduced pressure to give the corresponding 5-ring cyclic carbonate. All products were also confirmed by ^1^H- and ^13^C-NMR. For the recycling of the catalyst, after the first run, the catalyst was removed from the mixture by an external magnet and successfully washed with ethyl acetate (3 × 10 mL) and dichloromethane (2 × 10 mL) and dried under vacuum for 12 h and subsequently used for the next run.

### Synthesis of N-butyl dipicolinic carboxamide

The home-made pyridine 2,6-dicarbonyl chloride (0.5 g, 2.46 mmol) and dry THF (15 mL) were charged in a flame-dried balloon, then a mixture of butyl amine (0360 g, 0.487 mL, 4.93 mmol) and anhydrous pyridine (0.429 g, 0.436 ml, 5.42 mmol) in dry THF (5 mL) was added dropwise under argon. After having stirred at ambient temperature for 4 h, the reaction solution was filtered. THF and pyridine were removed by vacuum to obtain a white solid as N-butyl dipicolinic carboxamide^59^.

### Synthesis of homogeneous complex of La(III) with N-butyl dipicolinic carboxamide [Hom⋅La(III)]

In a typical procedure, to 5 ml of an ethanolic solution of LaCl_3_⋅7H_2_O (0.375 g, 1 mmol), N-butyl dipicolinic carboxamide (0.277 g, 1 mmol) was added and stirred at 80 °C for 15 h under argon. The product was collected by centrifugation, washed with ethanol, and dried at 80 °C to yield a light-yellow solid denoted as Hom⋅La(III)^59^.

### Carbon dioxide insertion studies

0.1 g of desired materials were stored under 1 bar CO_2_ pressure for 16 h at room temperature. After treatment, the samples were again weighted to determine the amount of captured CO_2_ in terms of mmol CO_2_ per gram of adsorbent^43^.

### Synthesis of Fe_3_O_4_@mSiO_2_

1 g of the as-prepared Fe_3_O_4_ was homogeneously dispersed in 200 mL of deionized water by ultrasonication for 15 min. The suspension was then added into a solution containing CTAB (1.5 g), deionized water (300 mL), ethanol (300 mL), and 28% ammonia solution (5.5 mL). A solution containing TEOS (3.33 mL, 15 mmol) was next added dropwise with stirring to the surfactant solution. The suspension was further stirred at room temperature for 6 h. The products were collected by an external magnet and washed several times with DI water and ethanol and dried at 80 °C overnight. To remove the CTAB template, 1 g of as-synthesized MON was stirred into a solution of ethanol (100 ml) and ammonium nitrate (0.2 mL) at 65 °C for 4 h. This process was repeated twice to completely remove all surfactants.

### Characterization methods

The pore structures of the prepared materials were observed by transmission electron microscopy (Philips CM-200) and were verified further by the nitrogen sorption analysis. N_2_ adsorption isotherms were measured at 77 K on Belsorp (BELMAX, Japan) analyzer using standard continuous procedures, and samples were first degassed at 353 K for 5 h. The specific surface area was determined from the linear part of the BET plot (P/P_0_ ≈ 0.05–0.15), the pore size distribution was calculated from the adsorption branch using Barrett–Joyner–Halenda (BJH) method, total pore volume was estimated based on the N_2_ adsorbed at P/P_0_ ≈ 0.995. Surface morphology of the materials was determined by a scanning electron microscope (SEM, Zeiss, Germany). Samples were deposited on a sample holder with an adhesive carbon foil and sputtered with gold. Elemental composition was characterized by an energy dispersive spectrometer (EDS) attached to the Zeiss-SEM. Powder X-ray diffraction patterns were carried out using a Siemens D-5000 diffractometer with CuKɑ (λ = 1.518 Å), a step size of 0.02° and counting time per step of 1.2 s, over arrange from 1° to 10°. Thermogravimetric analysis was performed by using a NETZSCH STA 409 PC/PG instrument at scan rates of 20 K min^−1^, with typically 5 mg sample under flowing O_2_. FT-IR spectra were recorded on a Brüker EQUINOX-55 instrument equipped with a liquid N_2_ cooled MCT detector. Magnetic properties were measured by using a vibrating sample magnetometer (Lake Shore, VSM 7400) with a maximum applied continuous field of 10 000 G at room temperature. Gas chromatography analyses were performed on Varian CP-3800 using a flame ionization detector (FID) using trimethylbenzene (TMB) as suitable internal standards. NMR spectra were recorded using a Brüker (^1^H frequency: 400 MHz, ^13^C frequency: 100 MHz).

## Supplementary Information

Supplementary Information.

## Data Availability

The datasets used and/or analysed during the current study available from the corresponding author on reasonable request.
