# Supplementary material for: Direct coupling of CO2 with epoxides catalyzed by lanthanum(III) supported on magnetic mesoporous organosilica nanoparticles
Source: Sci Rep. 2023 Apr 4;13:5521. doi: 10.1038/s41598-023-32647-9 (PMC10073222; doi:10.1038/s41598-023-32647-9)
Supplement: Supplementary file 1 — Supplementary Information. [file 41598_2023_32647_MOESM1_ESM.docx]

**Supplementary information for**

**Direct Coupling of CO_2_ with Epoxides Catalyzed by Lanthanum(III) Supported on Magnetic Mesoporous Organosilica Nanoparticles**

Kosar-Sadat Hoseini,^[a]^ Masoumeh Razaghi,^[a]^ Tohid Nouri^[a]^ and Mojtaba Khorasani*^[a,b]^

[a] Department of Chemistry, Institute for Advanced Studies in Basic Sciences (IASBS), No. 444, Prof. Yousef Sobouti Boulevard, Zanjan 45137-66731, Iran. E-mail: [m_khorasani@iasbs.ac.ir](mailto:m_khorasani@iasbs.ac.ir), Fax: +98-24-33153232; Tel: +98-24-3315-3223.

[b] Research Center for Basic Sciences & Modern Technologies (RBST), Institute for Advanced Studies in Basic Sciences, IASBS, Zanjan 45137-66731, Iran.

| **Table of Contents** | |
| --- | --- |
| **Title** | **Page** |
| 1. **Tables** ……………………………………………………………………………………………………………………………………………..……..….. | S3 |
| **Table S1**. Reusability results for La@MON catalyst in the coupling of CO_2_ with styrene oxide …………………...... | S3 |
| 2. **Figures** ……………………………………………………………………………………………………………………………………………….……… | S4 |
| **Figure S1**. Nitrogen adsorption-desorption of Re-La@MON ………………………………………………………….……..….……. | S4 |
| **Figure S2**. BJH Pore size distribution for Re-La@MON …………………………………………………………………………………... | S4 |
| **Figure S3**: FT-IR spectrum of Re-La@MON ……………………………………………………………………………………………..……… | S5 |
| **Figure S4**. TG pattern for Re-La@MON ………………………………………………………………………………………….……………… | S5 |
| **Figure S5.** FE-SEM image of Re-La@MON ……………………………………………………………………………………………..…..….. | S6 |
| **Figure S6.** ^1^H-NMR spectrum for 2,6-Pyridinedicarbonyl dichloride in CDCl_3_ as solvent ……………………….……….. | S6 |
| **Figure S7.** ^13^C-NMR spectrum for 2,6-Pyridinedicarbonyl dichloride in CDCl_3_ as solvent ……………………………….. | S7 |
| **Figure S8.** ^1^H-NMR spectrum for bis(3-(trimethoxysilyl)propyl)pyridine-2,6-dicarboxamide in CDCl_3_ as solvent | S7 |
| **Figure S9.** ^13^C-NMR spectrum for bis(3-(trimethoxysilyl)propyl)pyridine-2,6-dicarboxamide in CDCl_3_ as solvent | S8 |
| **Figure S10.** ^1^H-NMR spectrum for (chloromethyl)ethylene carbonate in CDCl_3_ as solvent ……………………………… | S8 |
| **Figure S11.** ^13^C-NMR spectrum for (chloromethyl)ethylene carbonate in CDCl_3_ as solvent …………………………… | S9 |
| **Figure S12.** ^1^H-NMR spectrum for (butoxymethyl)ethylene carbonate in CDCl_3_ as solvent ……………………..…… | S9 |
| **Figure S13.** ^13^C-NMR spectrum for (butoxymethyl)ethylene carbonate in CDCl_3_ as solvent ………………………..… | S10 |
| **Figure S14.** ^1^H-NMR spectrum for (isopropoxymethyl)ethylene carbonate in CDCl_3_ as solvent ………..…………… | S10 |
| **Figure S15.** ^13^C-NMR spectrum for (isopropoxymethyl)ethylene carbonate in CDCl_3_ as solvent …………….……… | S11 |
| **Figure S16.** ^1^H-NMR spectrum for (2-oxo-1,3-dioxolan-4-yl)-methyl methacrylate in CDCl_3_ as solvent …….…… | S11 |
| **Figure S17.** ^13^C-NMR spectrum for (2-oxo-1,3-dioxolan-4-yl)-methyl methacrylate in CDCl_3_ as solvent …..……. | S12 |
| **Figure S18.** ^1^H-NMR spectrum for 1,2-butylene carbonate in CDCl_3_ as solvent ………………………………………...…… | S12 |
| **Figure S19.** ^13^C-NMR spectrum for 1,2-butylene carbonate in CDCl_3_ as solvent ……………………………………..……… | S13 |
| **Figure S20**. ^1^H-NMR spectrum for cyclohexene carbonate in CDCl_3_ as solvent ……………………………………………… | S13 |
| **Figure S21**. ^13^C-NMR spectrum for cyclohexene carbonate in CDCl_3_ as solvent …………………………………............. | S14 |
| **Figure S22.** ^1^H-NMR spectrum for styrene carbonate in CDCl_3_ as solvent …………………………………………..…………. | S14 |
| **Figure S23.** ^13^C-NMR spectrum for styrene carbonate in CDCl_3_ as solvent ………………………………………..…………… | S15 |
| **Figure S24.** ^1^H-NMR spectrum for (phenoxymethyl ) ethylene carbonate in CDCl_3_ as solvent …………..………….. | S15 |
| **Figure S25.** ^13^C-NMR spectrum for (phenoxymethyl ) ethylene carbonate in CDCl_3_ as solvent …………..…………. | S16 |
| **Figure S26.** ^1^H-NMR spectrum for propyl carbonate in CDCl_3_ as solvent ……………………………………..………………… | S16 |
| **Figure S27.** ^13^C-NMR spectrum for propyl carbonate in CDCl_3_ as solvent ……………………………………..……………….. | S17 |
| **Figure S28.** ^1^H-NMR spectrum for glycidol carbonate in CDCl_3_ as solvent …………………………………..…………………. | S17 |
| **Figure S29.** ^13^C-NMR spectrum for glycidol carbonate in CDCl_3_ as solvent …………………………………..………………… | S18 |
| **Figure S30.** ^1^H-NMR spectrum for allyl glycidyl carbonate in CDCl_3_ as solvent ………………………………………………. | S18 |
| **Figure S31.** ^13^C-NMR spectrum for allyl glycidyl carbonate in CDCl_3_ as solvent ……………………………………………… | S19 |
| **Figure S32.** ^1^H-NMR spectrum for N-butyl dipicolinic carboxamide in CDCl_3_ as solvent …………………..……………. | S19 |
| **Figure S33.** ^13^C-NMR spectrum for N-butyl dipicolinic carboxamide in CDCl_3_ as solvent …………………….…………. | S20 |
| **Figure S34.** ^1^H-NMR spectrum for complex of La(III) with N-butyl dipicolinic carboxamide in DMSO-d_6_ ……….. | S20 |

**1. Tables.**

| **Table S1.** Reusability results for La@MON catalyst in the coupling of CO_2_ with styrene oxide.^[a]^ | |
| --- | --- |
|  | |
| Run | Yield (%)^[b]^ |
| 1 | 92 |
| 2 | 92 |
| 3 | 91 |
| 4 | 92 |
| 5 | 88 |
| [a]Reaction conditions: Styrene oxide (10 mmol), La@MON (230 mg), TBAI (0.5 mol%) and CO_2_ (10 Bar) at 100 °C within 5 h.  [b] GC yield by using TMB internal standard. | |

1. **Figures**


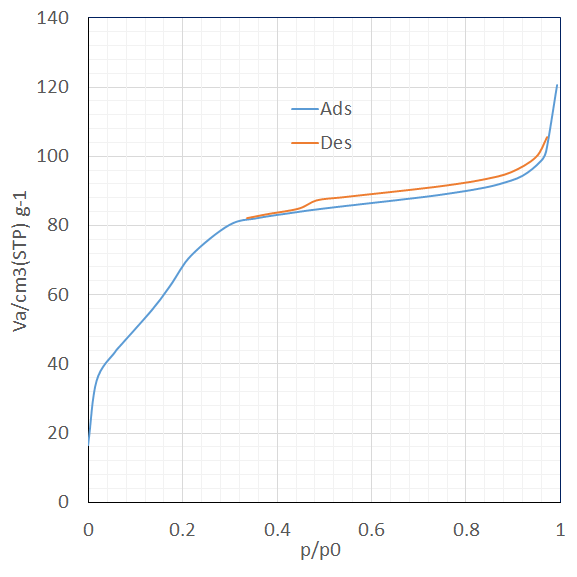


**Figure S1**. Nitrogen adsorption-desorption of Re-La@MON


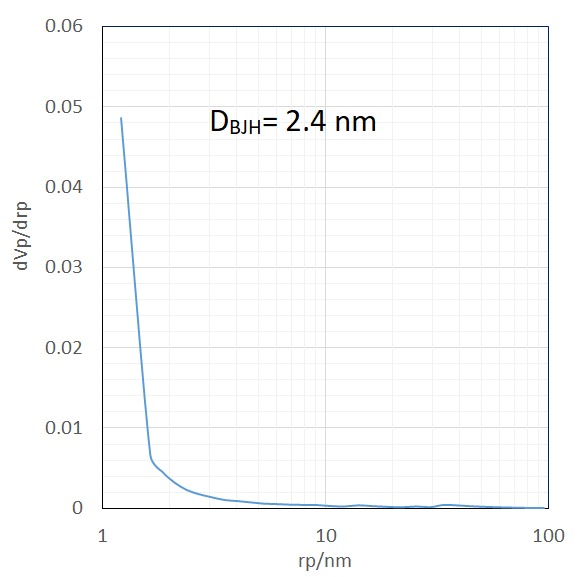


**Figure S2**. BJH pore size distribution of Re-La@MON

**
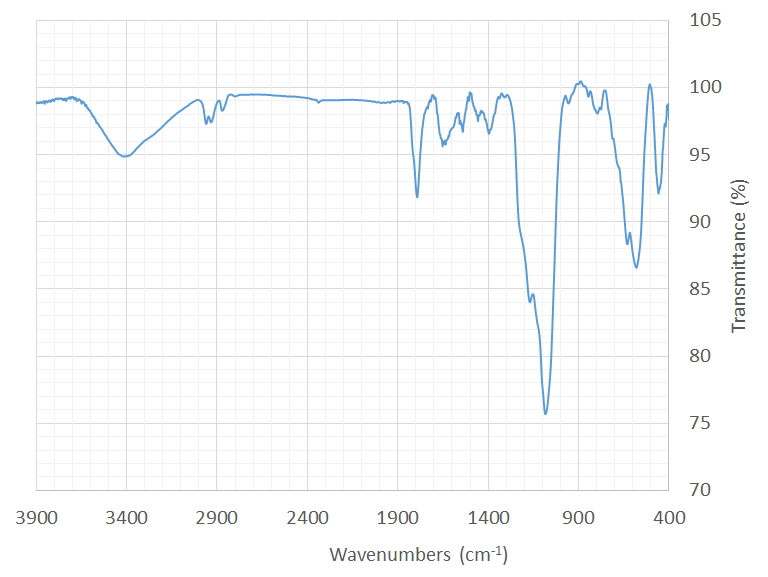
**

**Figure S3**. FT-IR spectrum of Re-La@MON





**Figure S4**. TG pattern for Re-La@MON


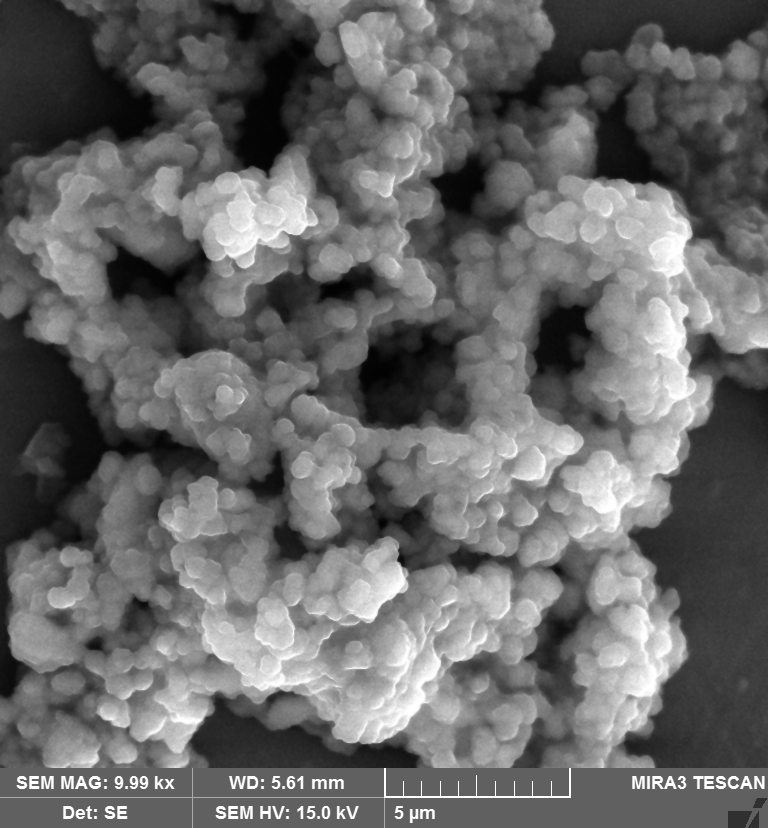


**Figure S5**. FE-SEM image of Re-La@MON


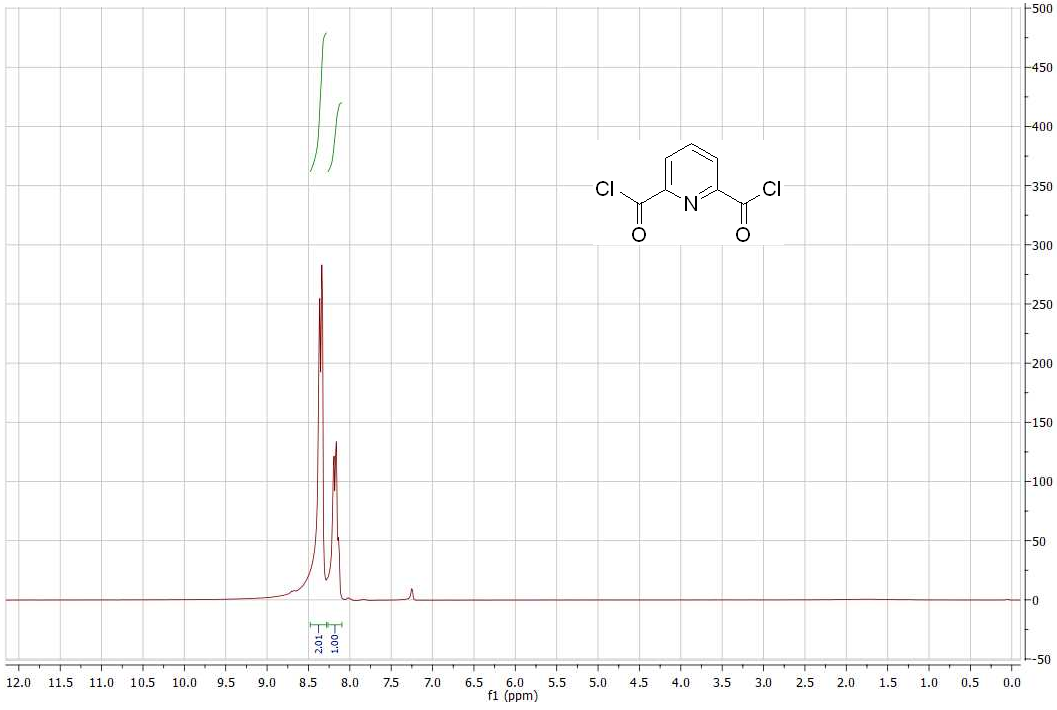


**Figure S6.** ^1^H-NMR spectrum for 2,6-Pyridinedicarbonyl dichloride in CDCl_3_ as solvent


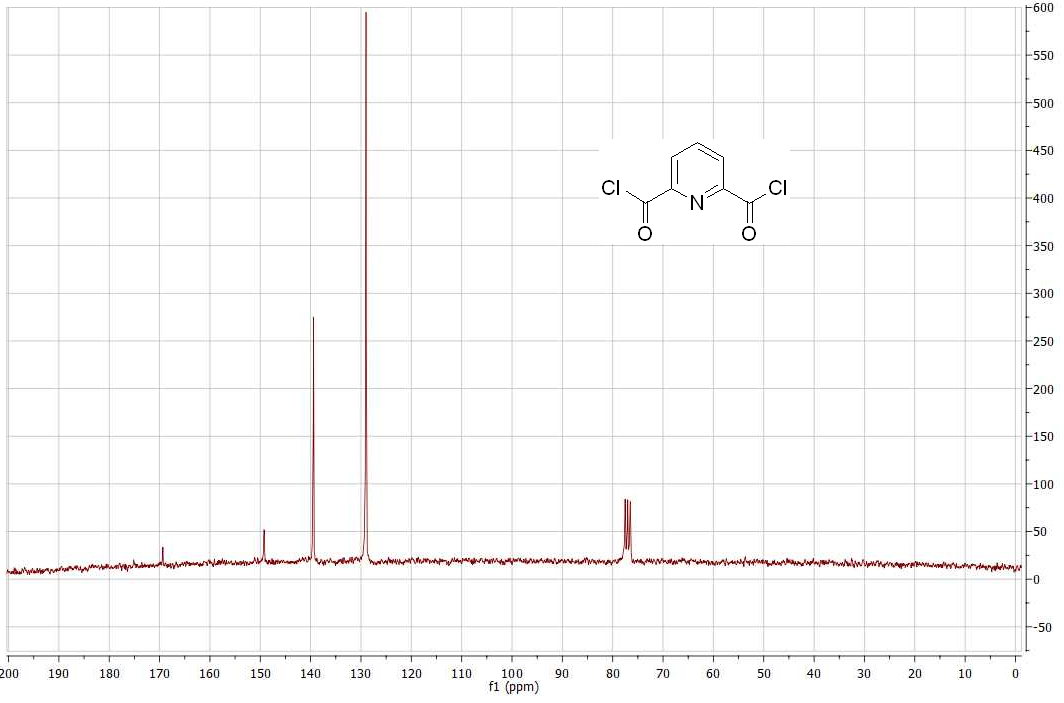


**Figure S7.** ^13^C-NMR spectrum for 2,6-Pyridinedicarbonyl dichloride in CDCl_3_ as solvent


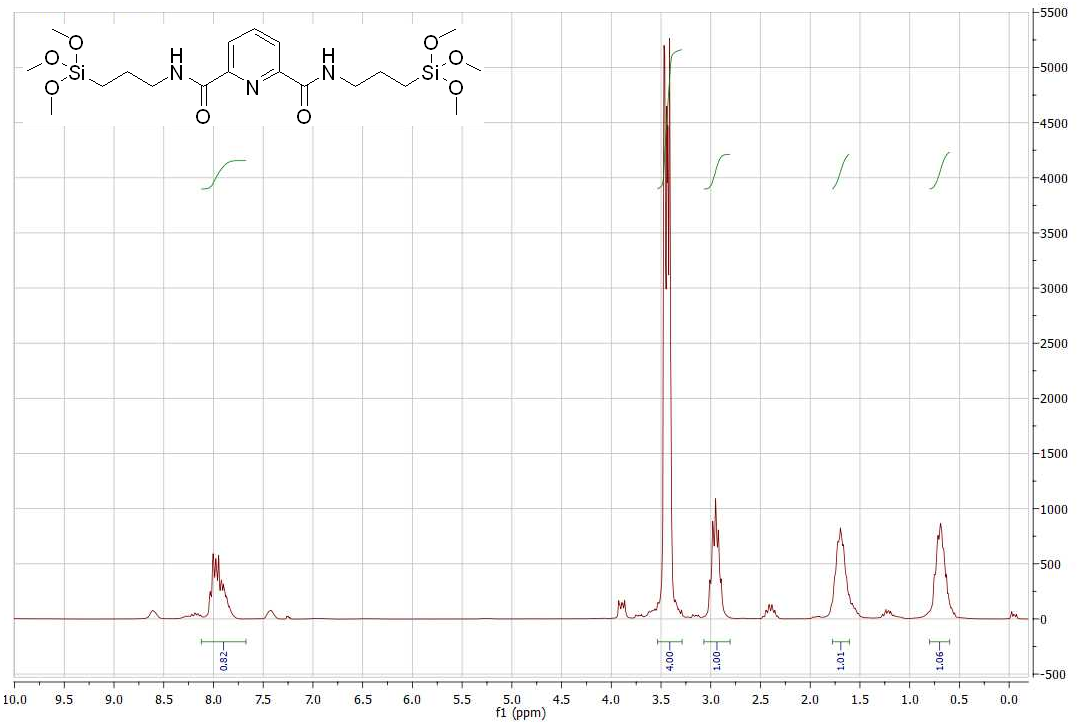


**Figure S8.** ^1^H-NMR spectrum for bis(3-(trimethoxysilyl)propyl)pyridine-2,6-dicarboxamide in CDCl_3_ as solvent


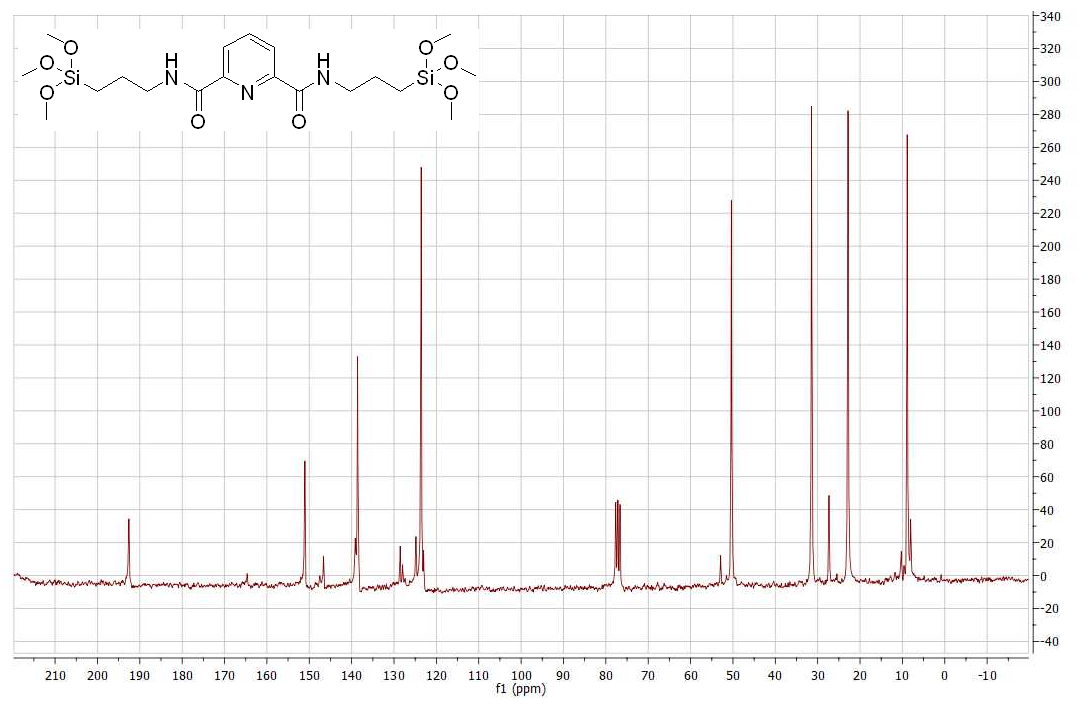


**Figure S9.** ^13^C-NMR spectrum for bis(3-(trimethoxysilyl)propyl)pyridine-2,6-dicarboxamide in CDCl_3_ as solvent


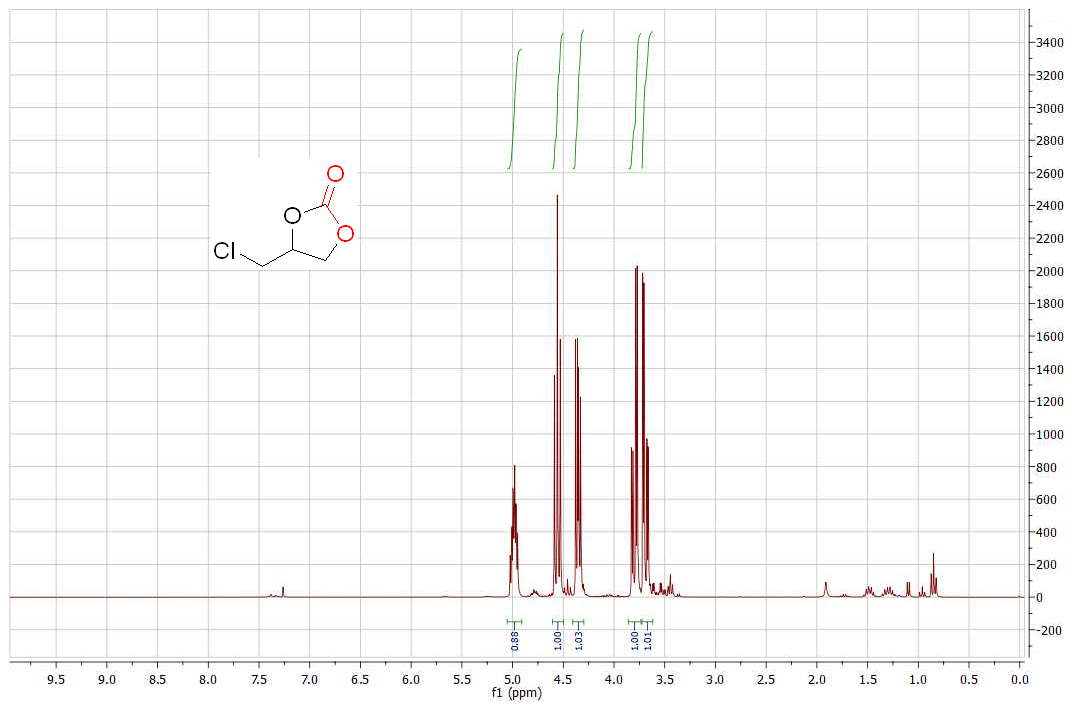


**Figure S10.** ^1^H-NMR spectrum for (chloromethyl)ethylene carbonate in CDCl_3_ as solvent


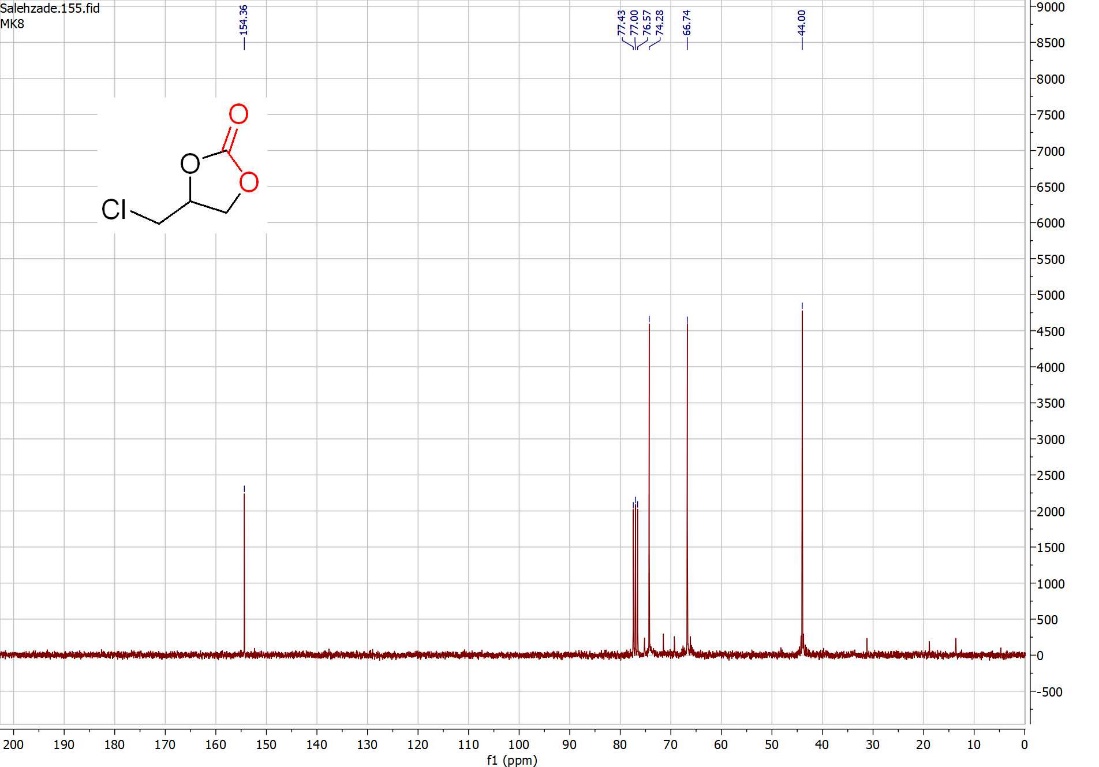


**Figure S11.** ^13^C-NMR spectrum for (chloromethyl)ethylene carbonate in CDCl_3_ as solvent


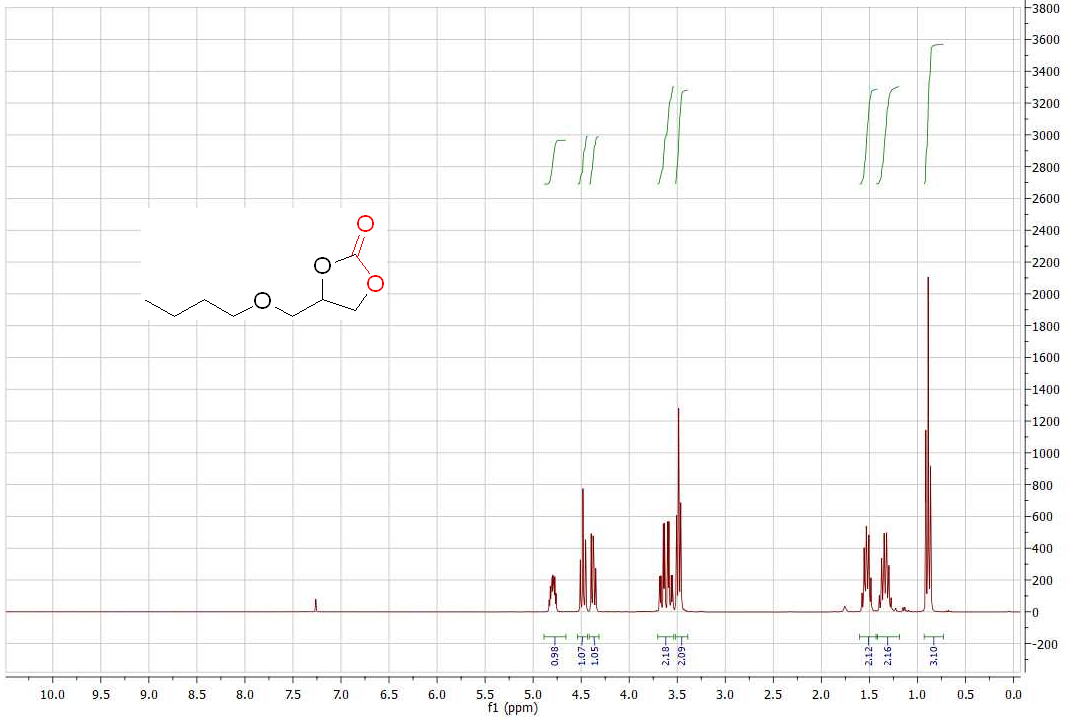


**Figure S12.** ^1^H-NMR spectrum for (butoxymethyl)ethylene carbonate in CDCl_3_ as solvent


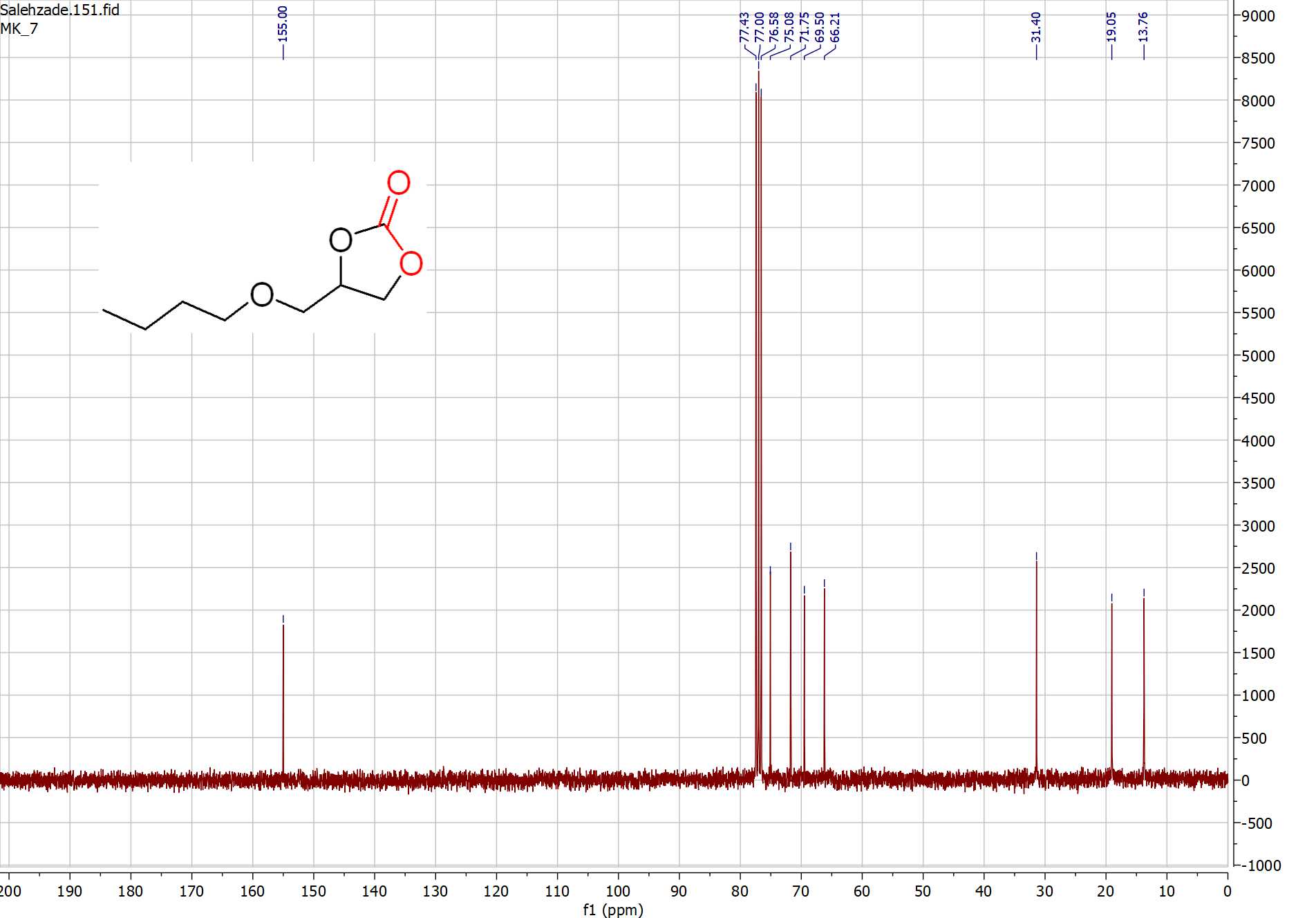


**Figure S13.** ^13^C-NMR spectrum for (butoxymethyl)ethylene carbonate in CDCl_3_ as solvent


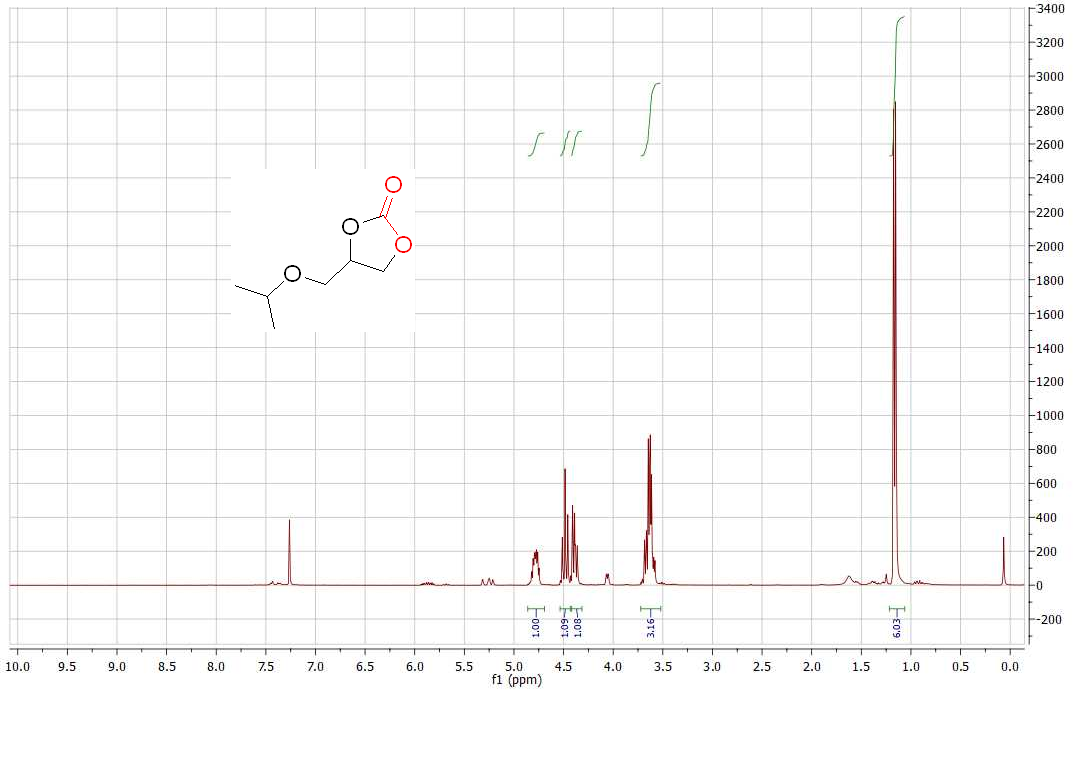


**Figure S14.** ^1^H-NMR spectrum for (isopropoxymethyl)ethylene carbonate in CDCl_3_ as solvent


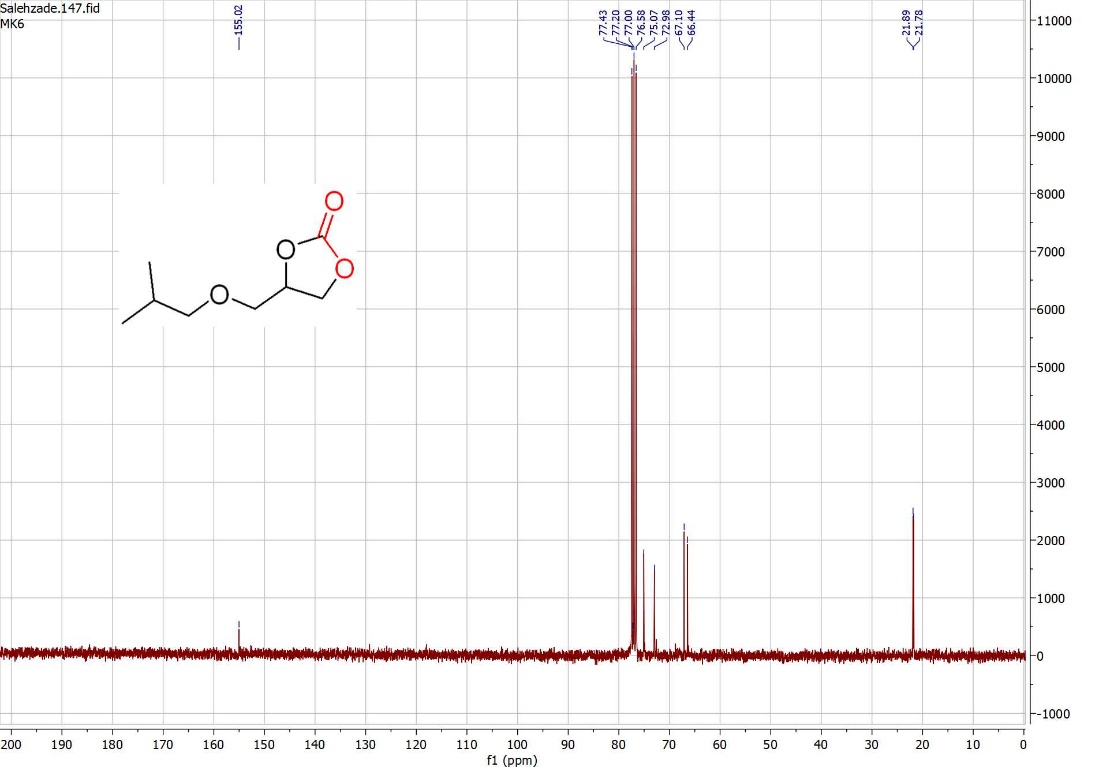


**Figure S15.** ^13^C-NMR spectrum for (isopropoxymethyl)ethylene carbonate in CDCl_3_ as solvent


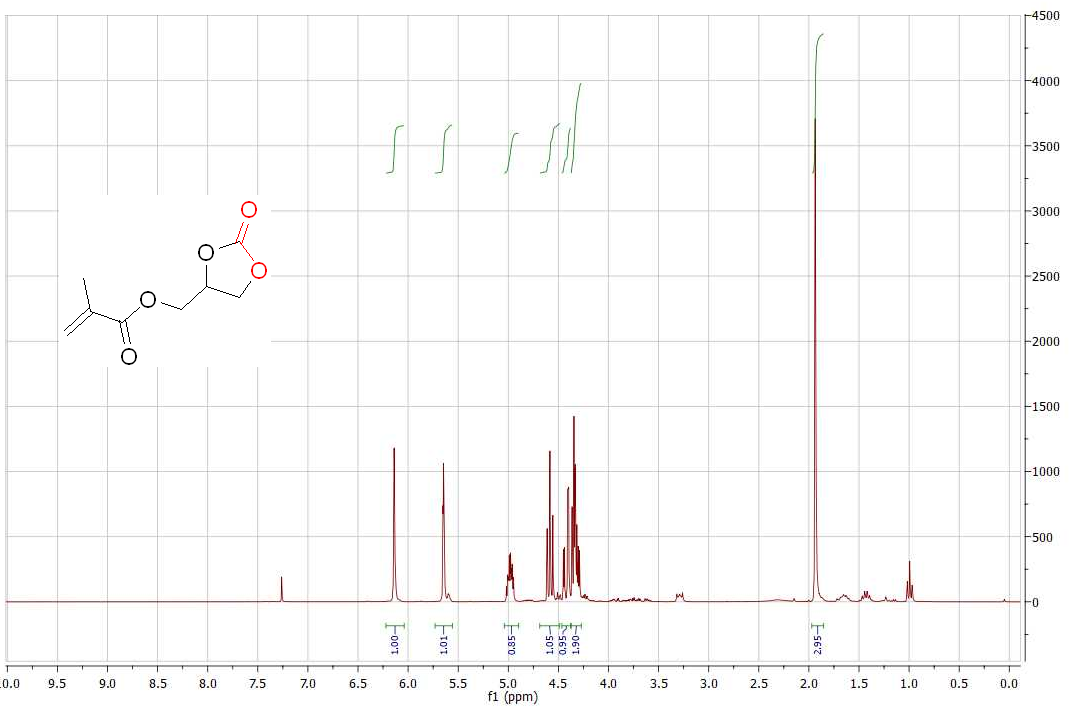


**Figure S16.** ^1^H-NMR spectrum for (2-oxo-1,3-dioxolan-4-yl)-methyl methacrylate in CDCl_3_ as solvent


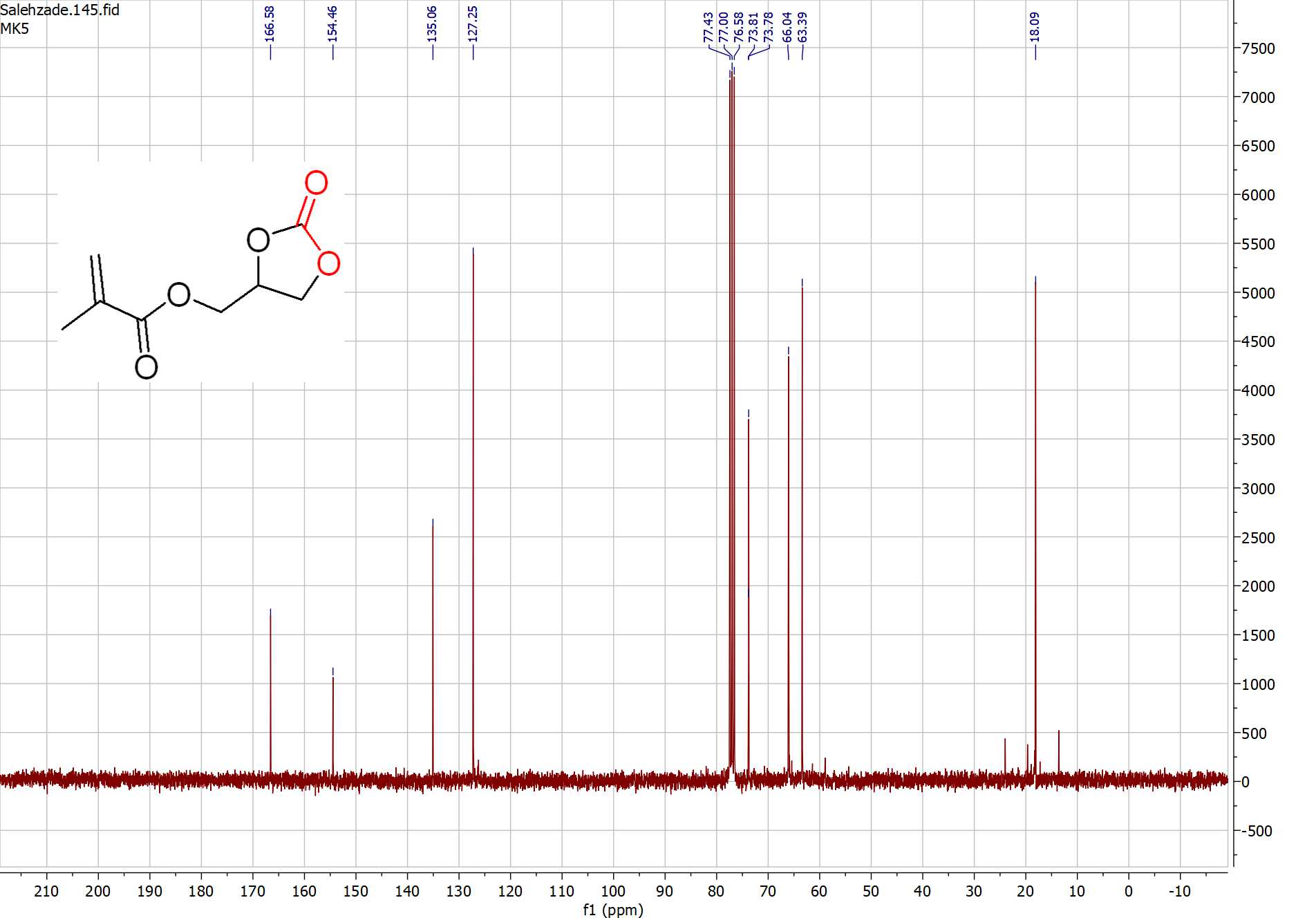


**Figure S17.** ^13^C-NMR spectrum for (2-oxo-1,3-dioxolan-4-yl)-methyl methacrylate in CDCl_3_ as solvent


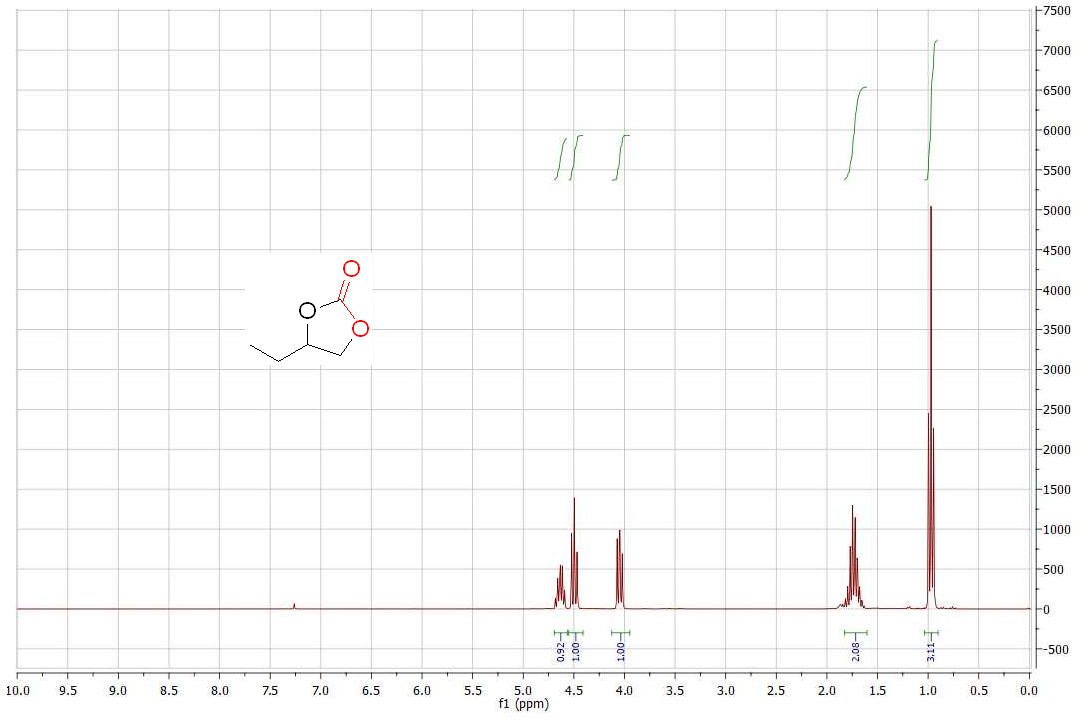


**Figure S18.** ^1^H-NMR spectrum for 1,2-butylene carbonate in CDCl_3_ as solvent


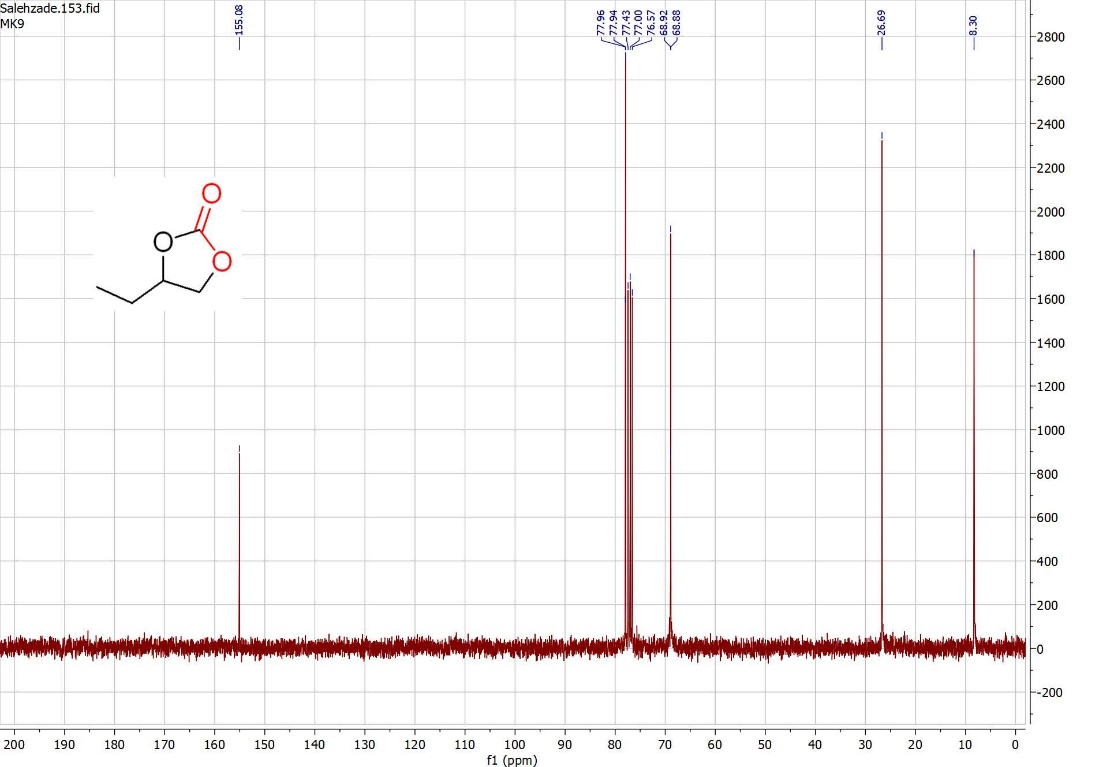
c

**Figure S19.** ^13^C-NMR spectrum for 1,2-butylene carbonate in CDCl_3_ as solvent


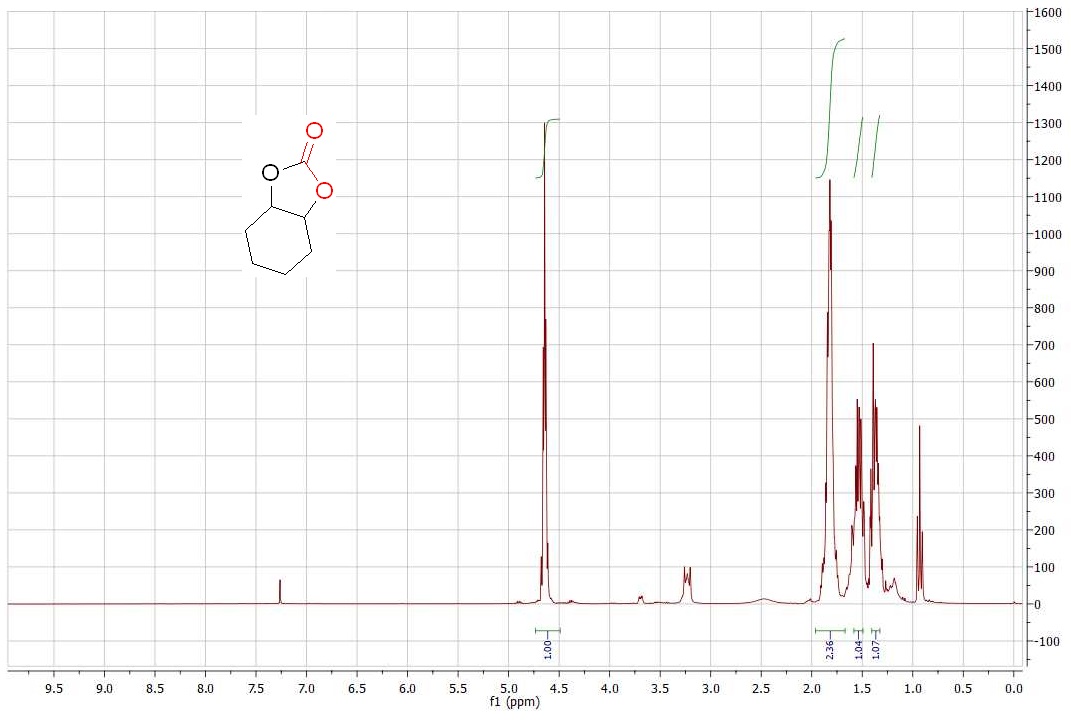


**Figure S20**. ^1^H-NMR spectrum for cyclohexene carbonate in CDCl_3_ as solvent


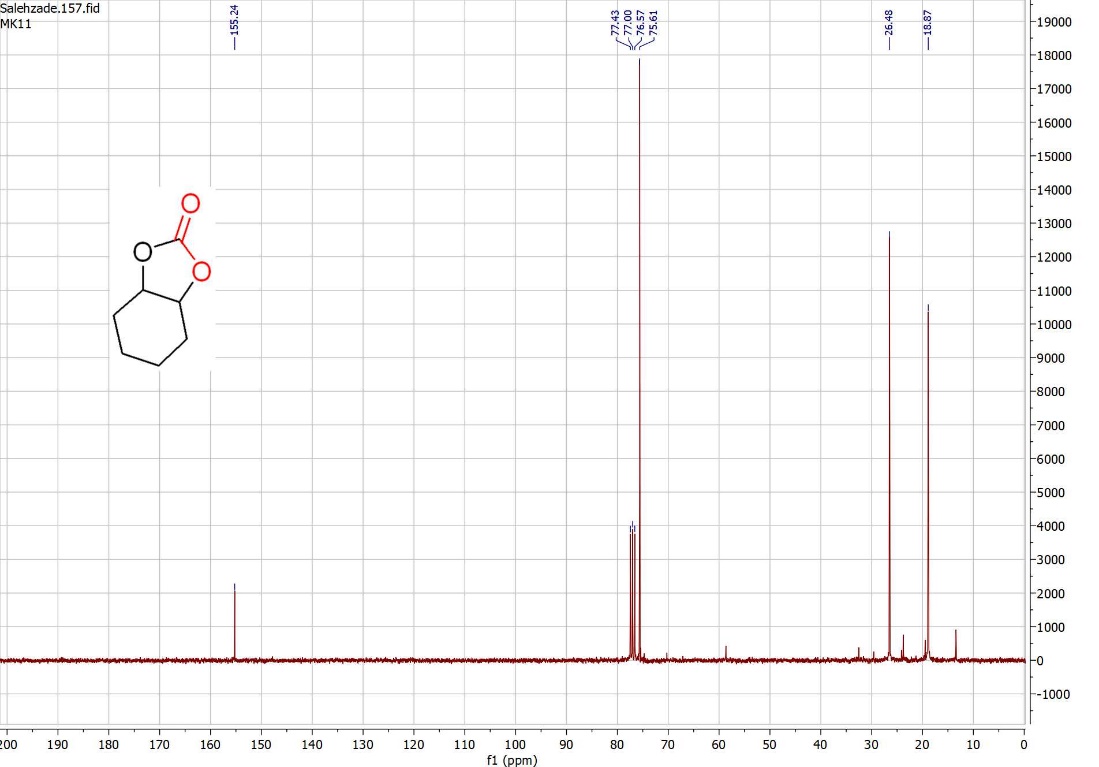


**Figure S21**. ^13^C-NMR spectrum for cyclohexene carbonate in CDCl_3_ as solvent


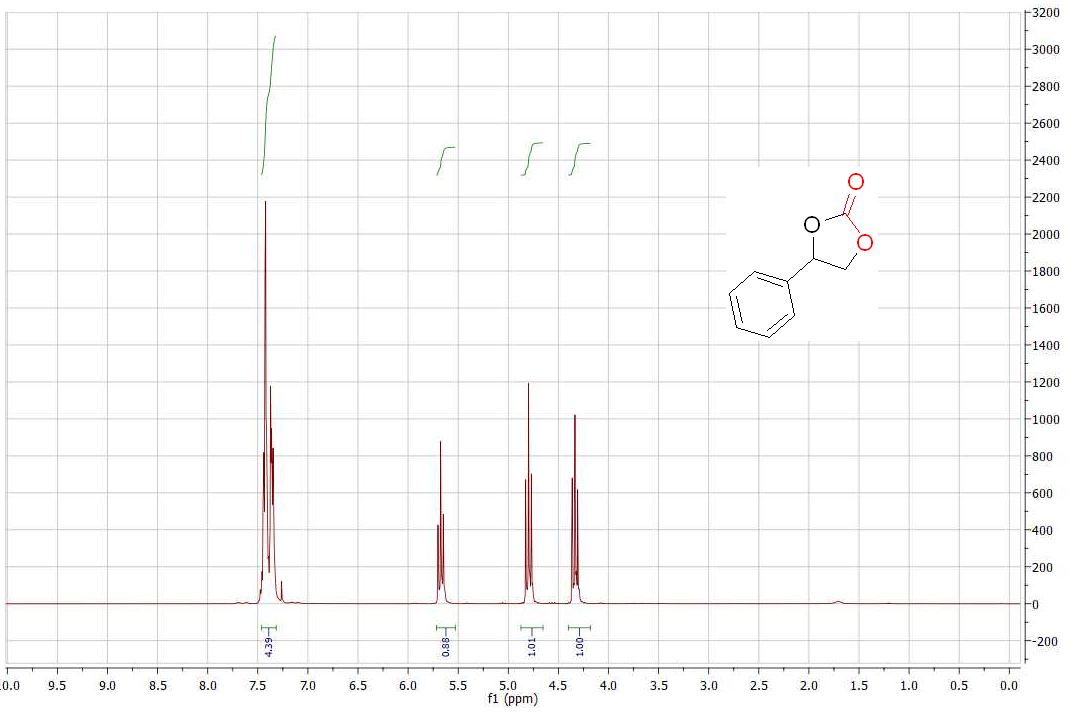


**Figure S22.** ^1^H-NMR spectrum for styrene carbonate in CDCl_3_ as solvent


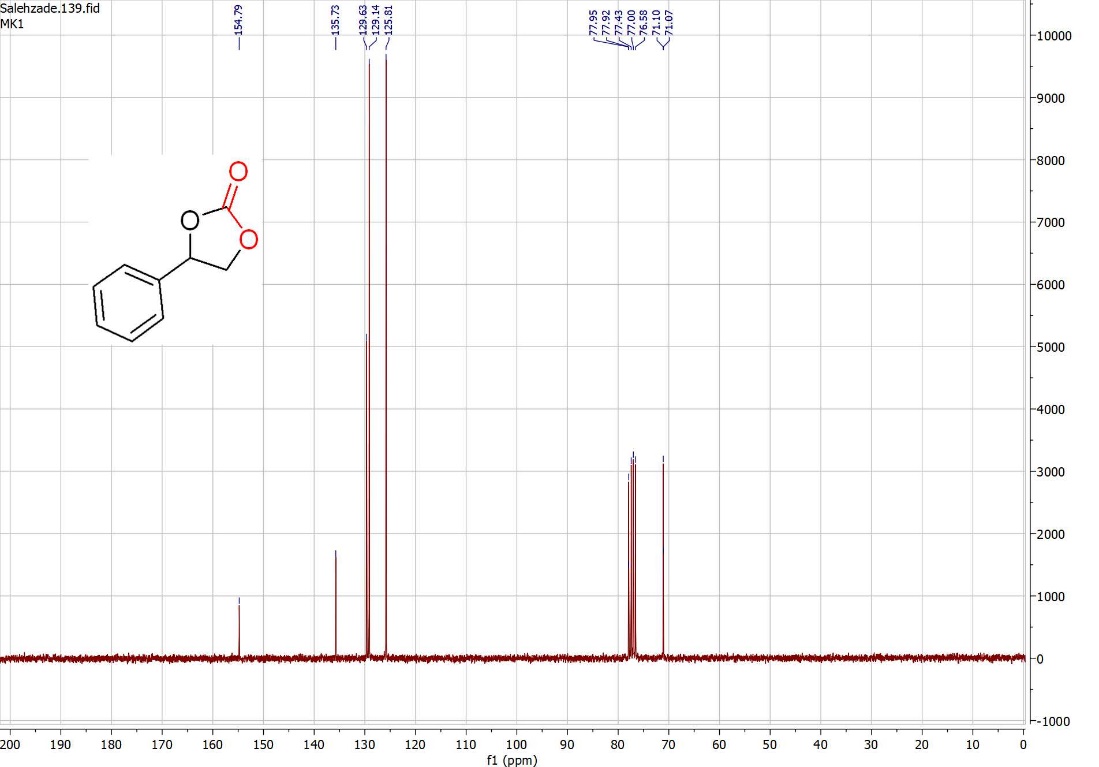


**Figure S23.** ^13^C-NMR spectrum for styrene carbonate in CDCl_3_ as solvent


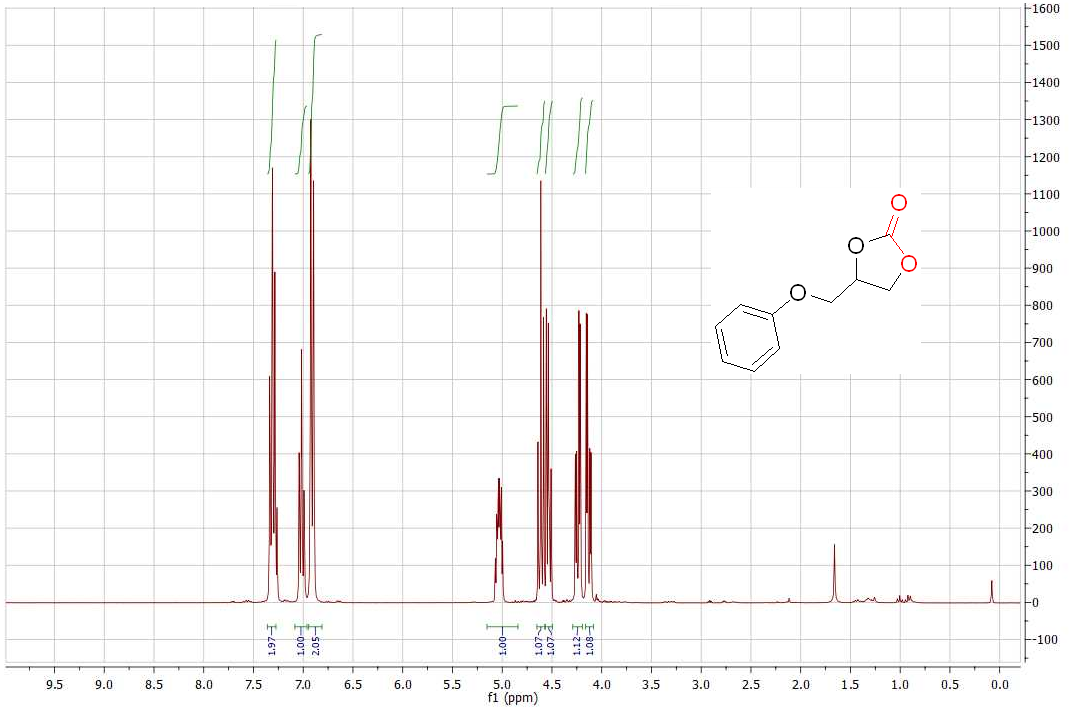


**Figure S24.** ^1^H-NMR spectrum for (phenoxymethyl ) ethylene carbonate in CDCl_3_ as solvent


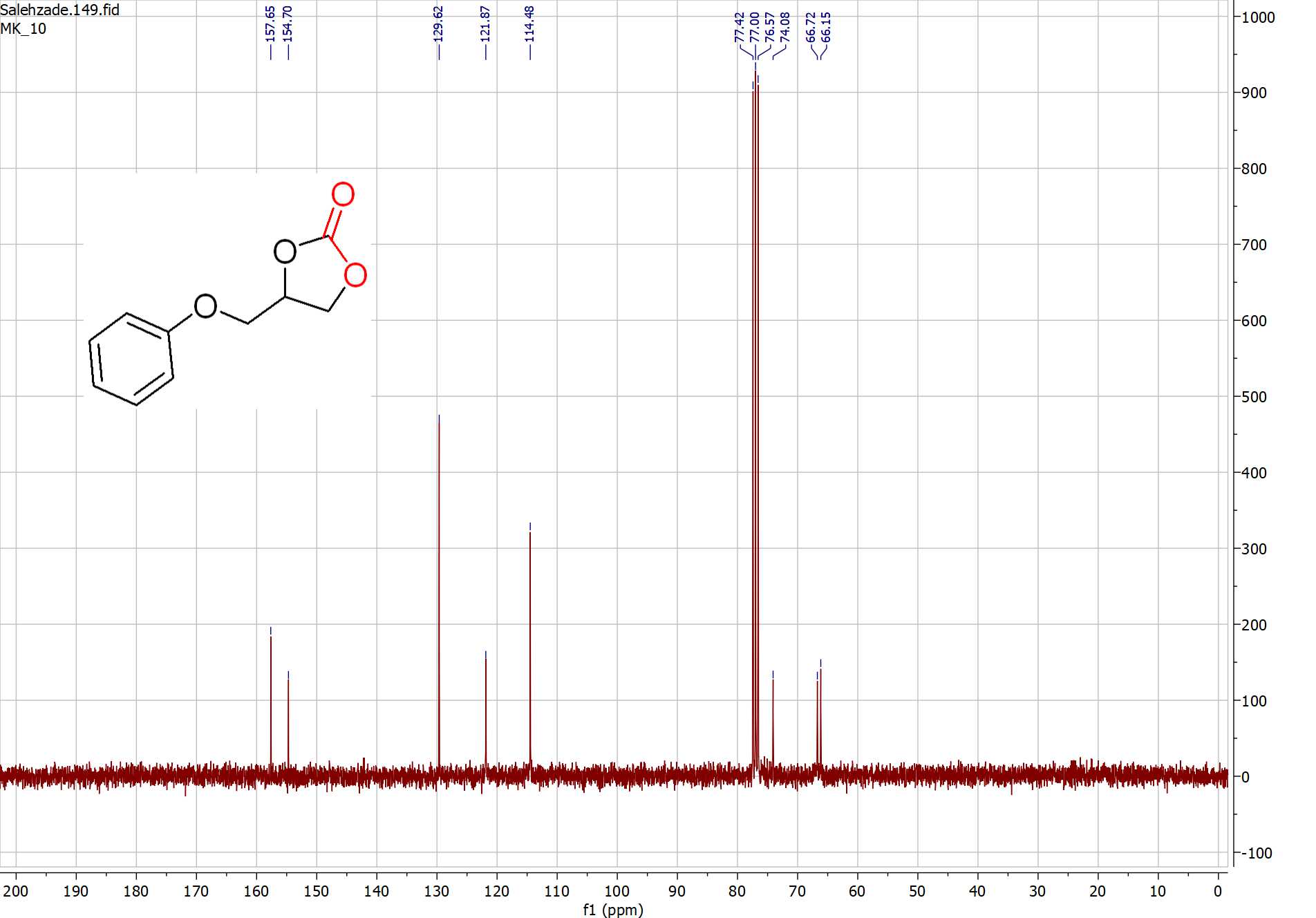


**Figure S25.** ^13^C-NMR spectrum for (phenoxymethyl ) ethylene carbonate in CDCl_3_ as solvent


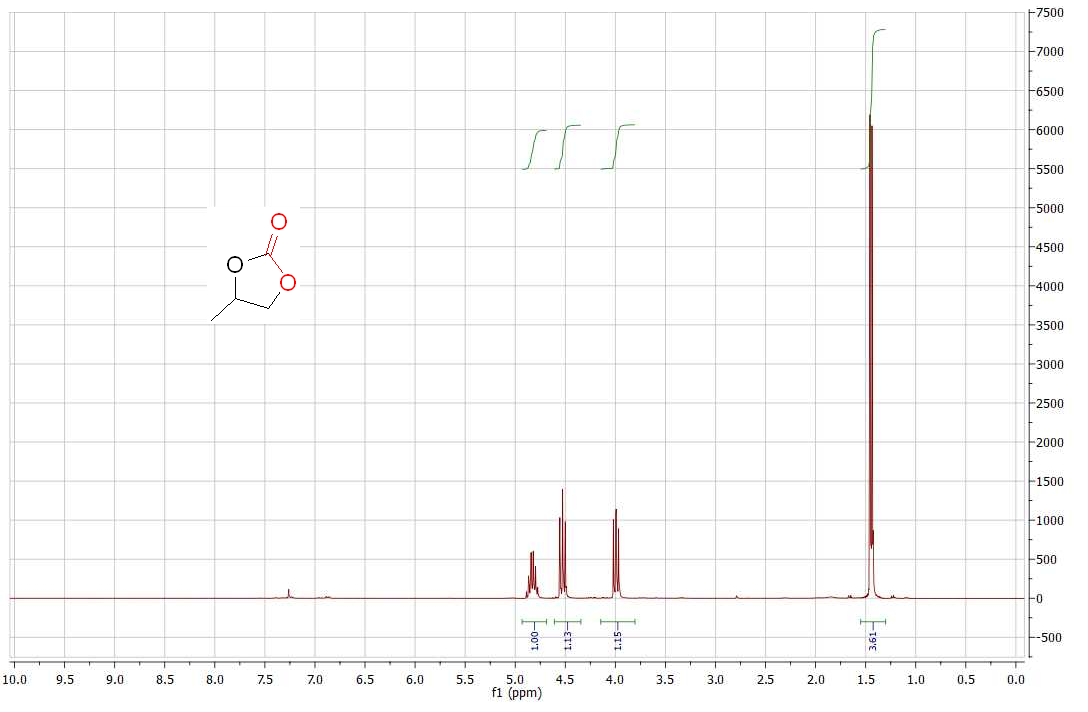


**Figure S26.** ^1^H-NMR spectrum for propyl carbonate in CDCl_3_ as solvent


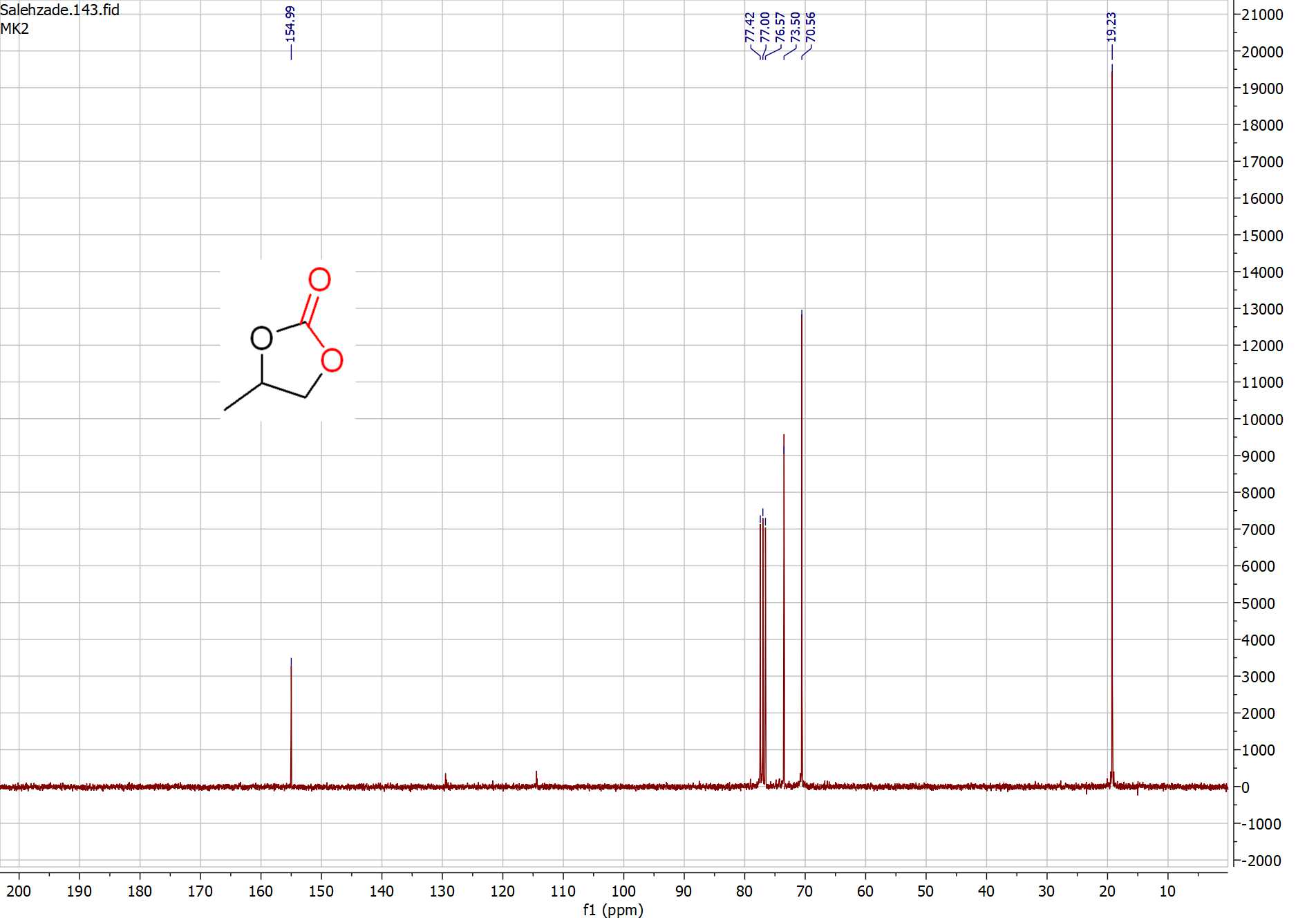


**Figure S27.** ^13^C-NMR spectrum for propyl carbonate in CDCl_3_ as solvent


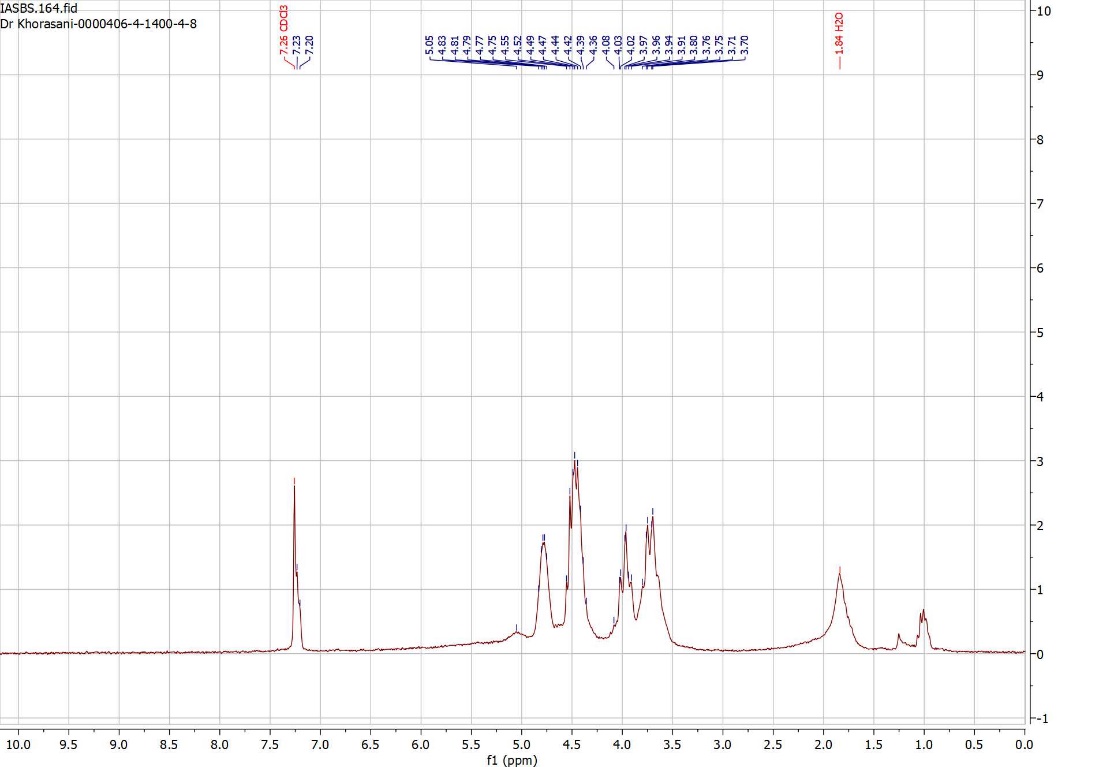


**Figure S28.** ^1^H-NMR spectrum for glycidol carbonate in CDCl_3_ as solvent


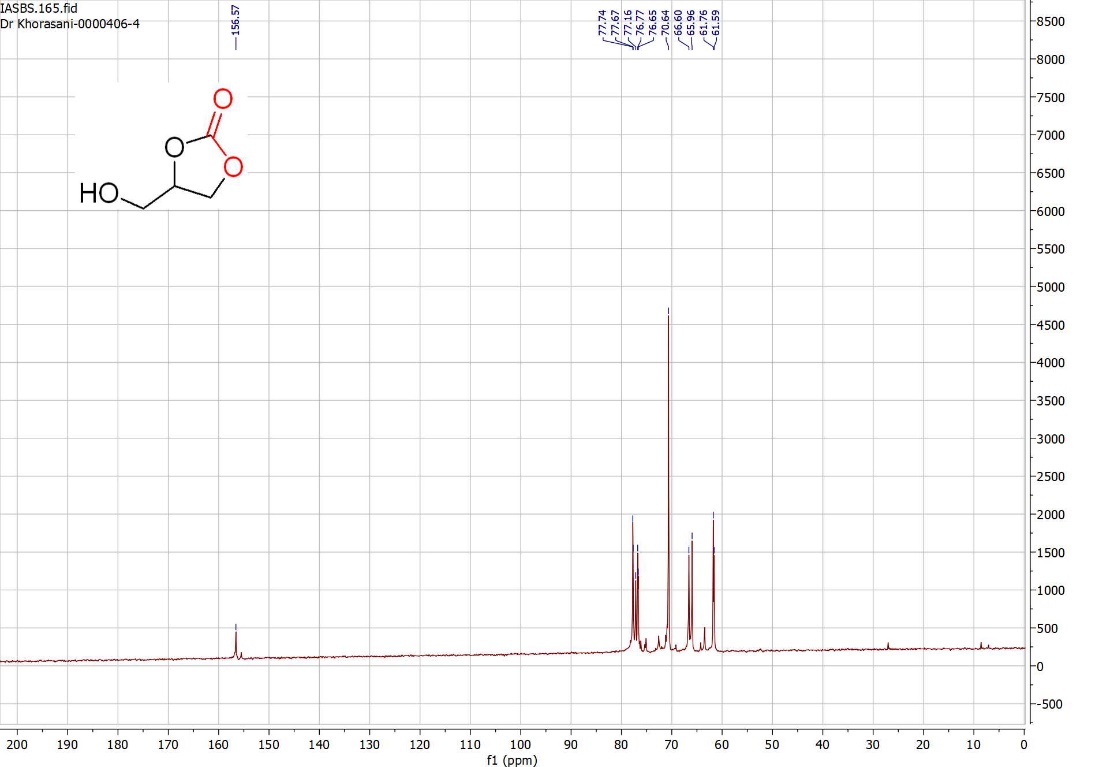


**Figure S29.** ^13^C-NMR spectrum for glycidol carbonate in CDCl_3_ as solvent


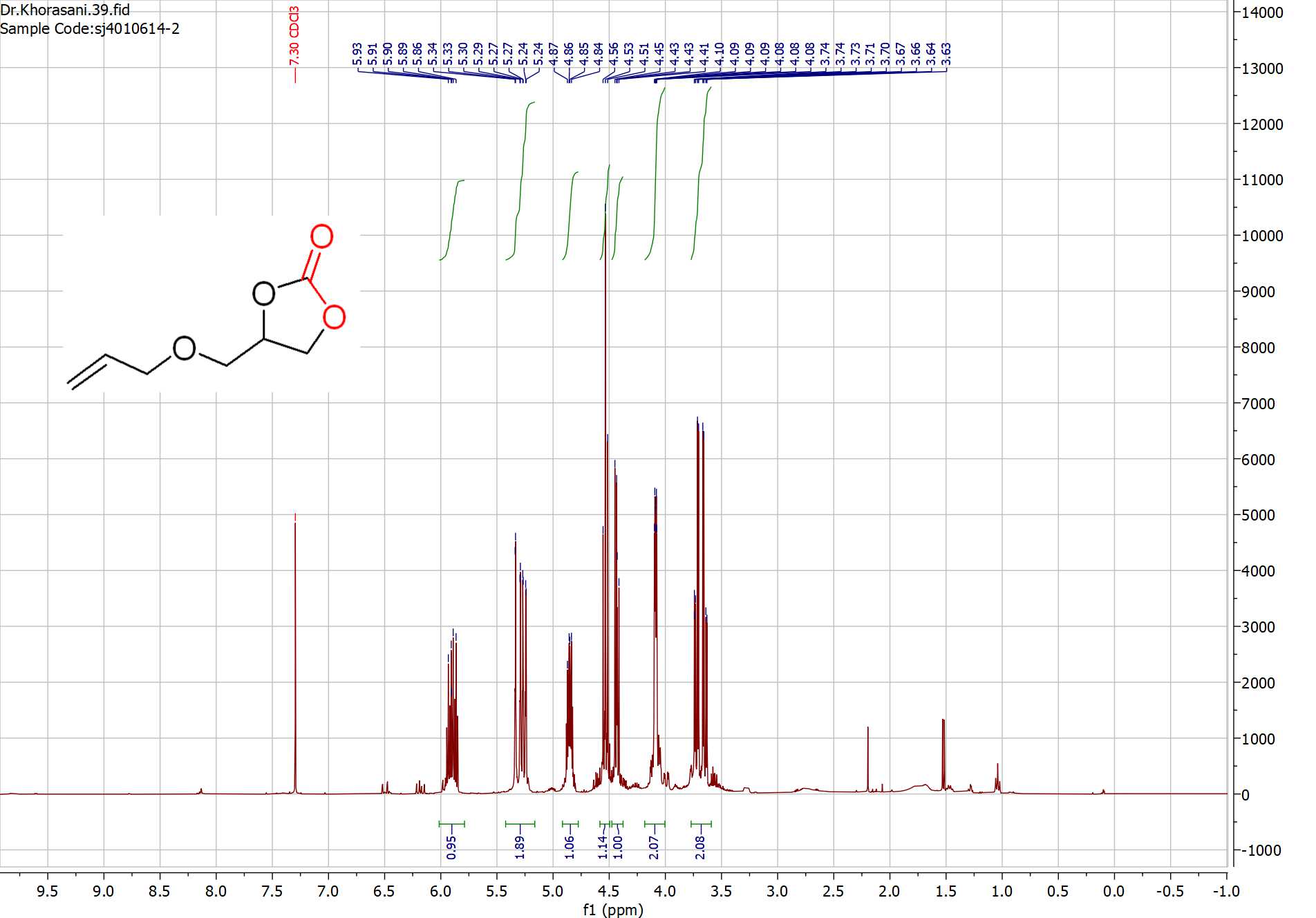


**Figure S30.** ^1^H-NMR spectrum for allyl glycidyl carbonate in CDCl_3_ as solvent


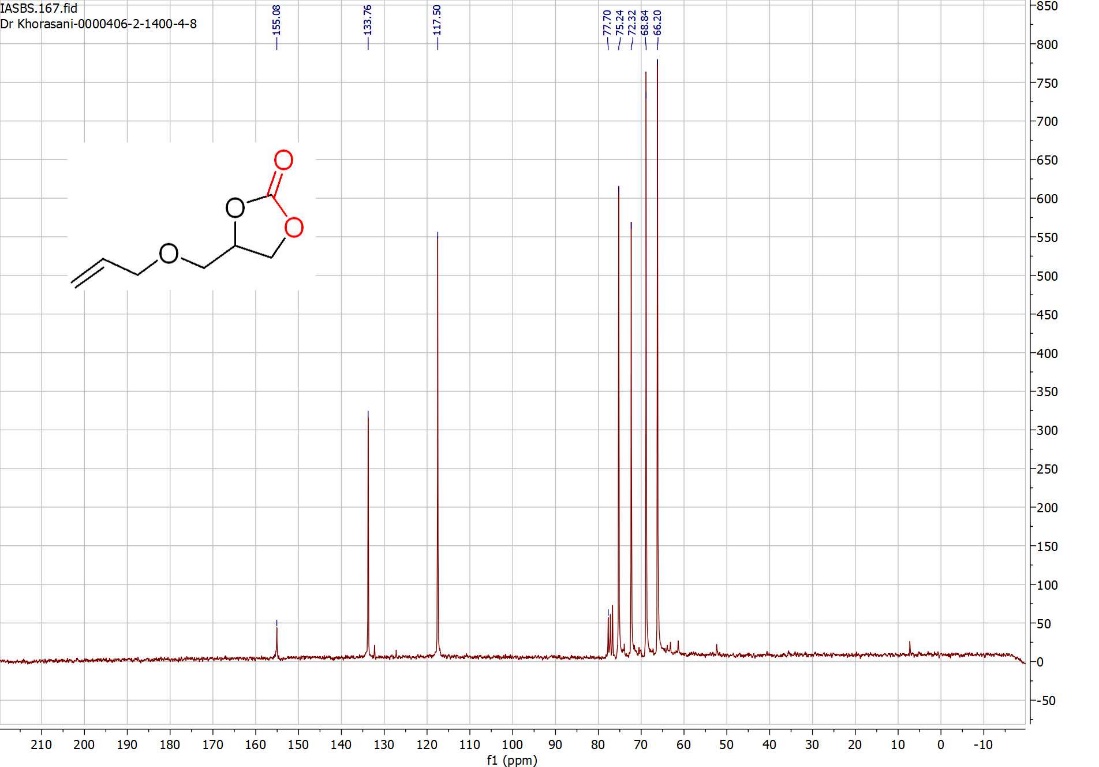


**Figure S31.** ^13^C-NMR spectrum for allyl glycidyl carbonate in CDCl_3_ as solvent


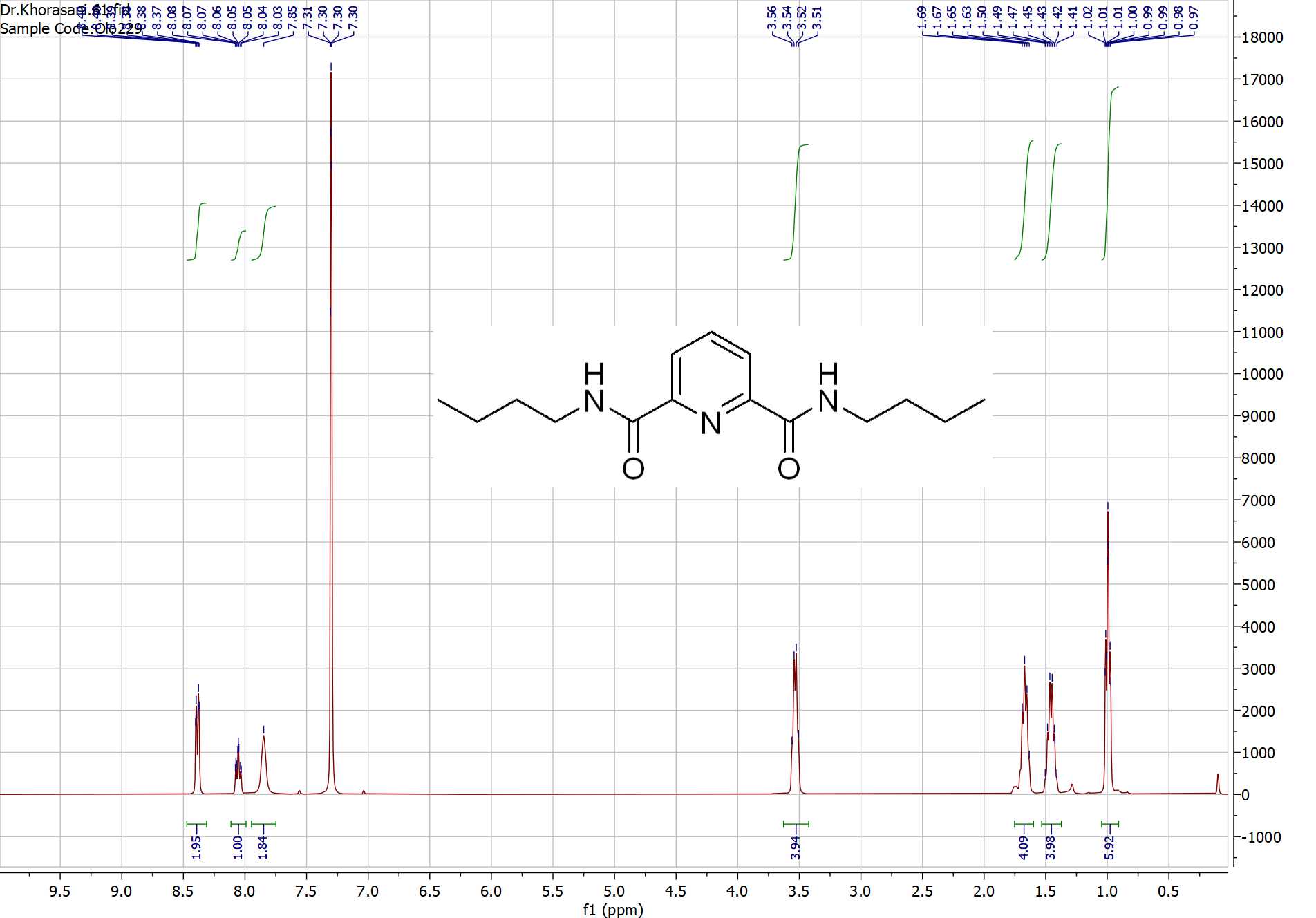


**Figure S32.** ^1^H-NMR spectrum for N-butyl dipicolinic carboxamide in CDCl_3_ as solvent


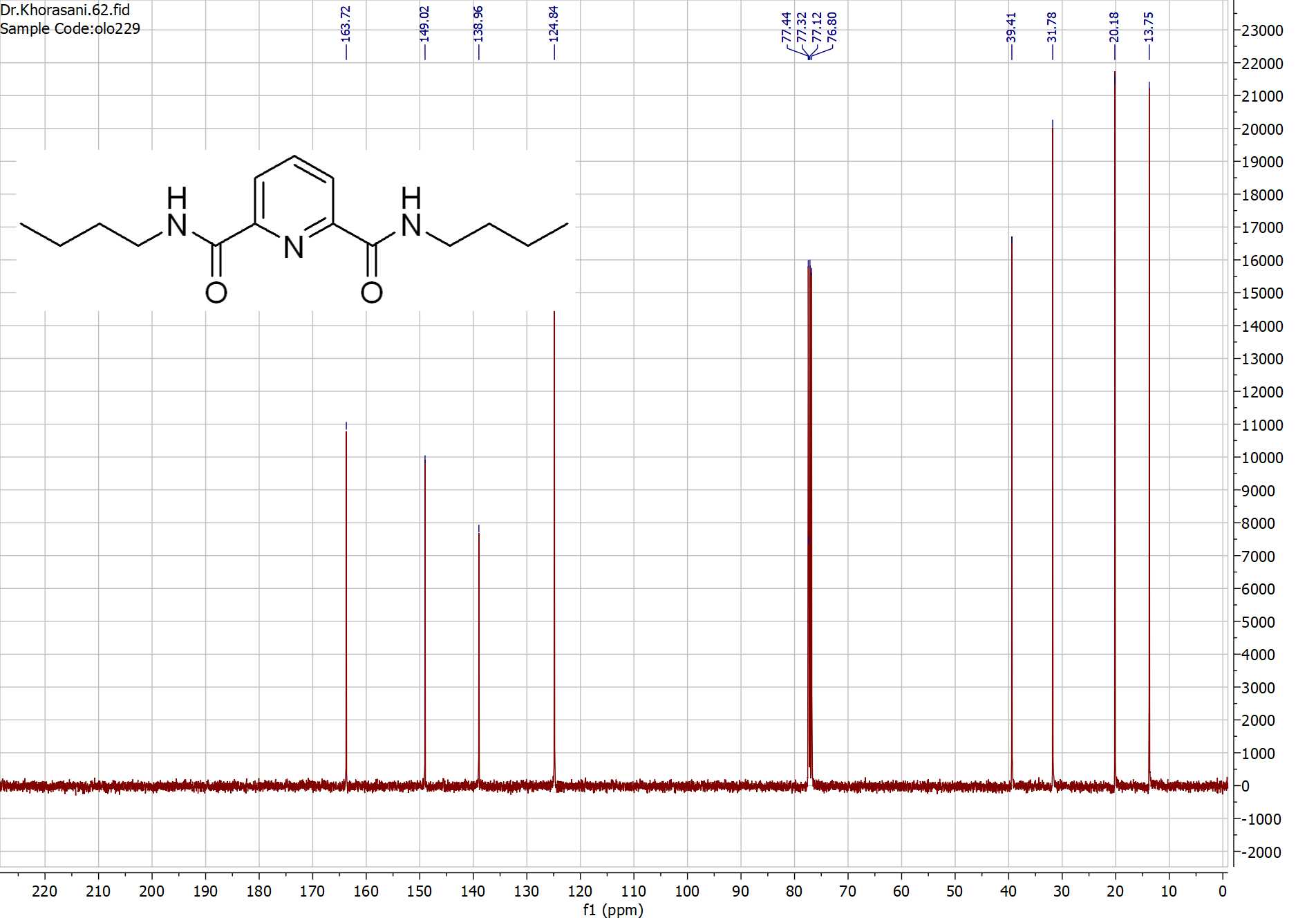


**Figure S33.** ^13^C-NMR spectrum for N-butyl dipicolinic carboxamide in CDCl_3_ as solvent


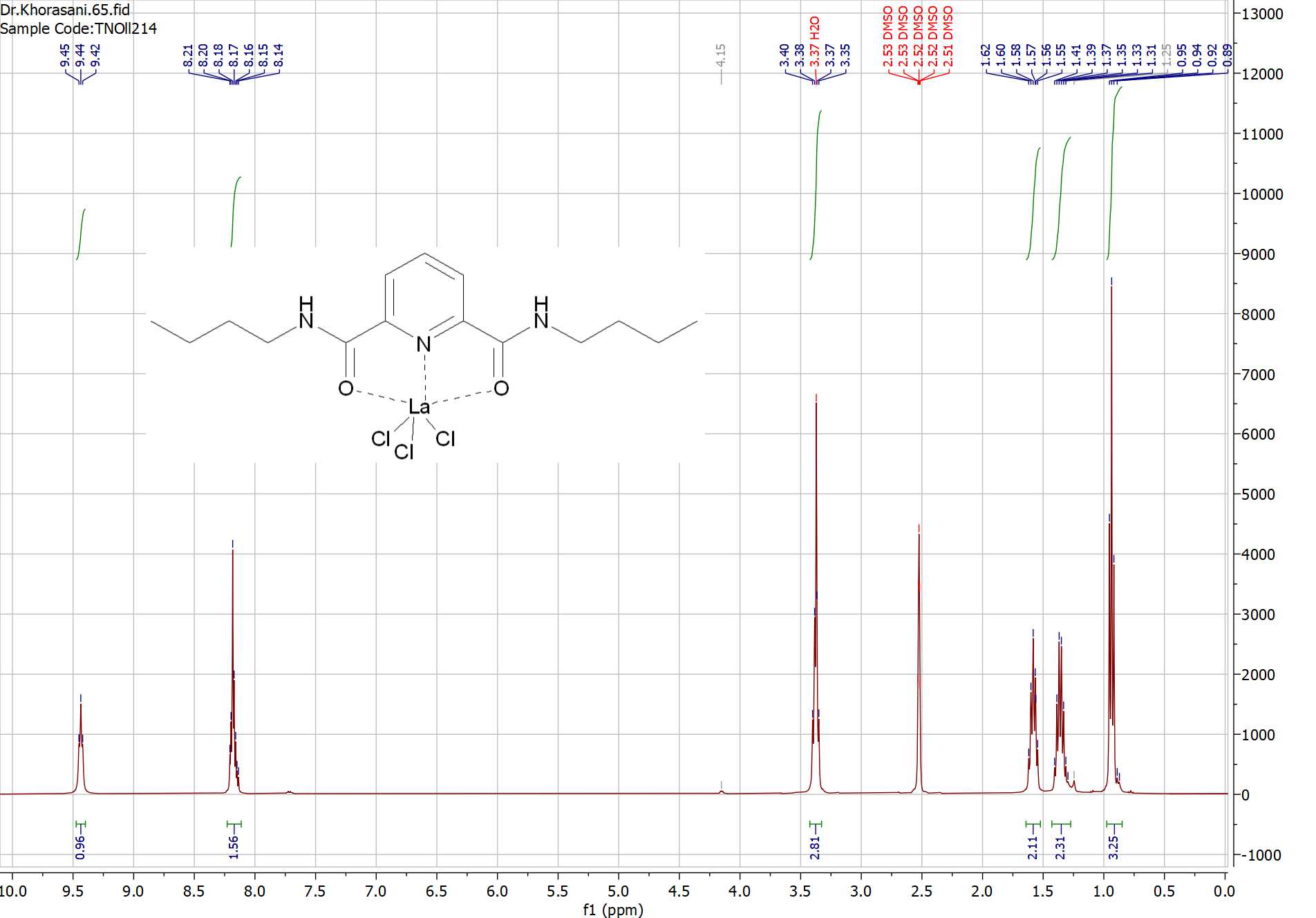


**Figure S34.** ^1^H-NMR spectrum for complex of La(III) with N-butyl dipicolinic carboxamide in DMSO-d_6_ as solvent
